# Immunomodulation of Skin Repair: Cell-Based Therapeutic Strategies for Skin Replacement (A Comprehensive Review)

**DOI:** 10.3390/biomedicines10010118

**Published:** 2022-01-06

**Authors:** Shima Tavakoli, Marta A. Kisiel, Thomas Biedermann, Agnes S. Klar

**Affiliations:** 1 Division of Polymer Chemistry, Department of Chemistry-Ångstrom Laboratory, Uppsala University, 75121 Uppsala, Sweden; shima.tavakoli@kemi.uu.se; 2 Environmental and Occupational Medicine, Medical Sciences, Uppsala University Hospital, 75237 Uppsala, Sweden; marta.kisiel@medsci.uu.se; 3 Tissue Biology Research Unit, University Children’s Hospital Zurich, University of Zurich, 8952 Schlieren, Switzerland; Thomas.Biedermann@kispi.uzh.ch; 4 Children’s Research Center, University Children’s Hospital Zurich, 8032 Zurich, Switzerland

**Keywords:** biomaterials, skin substitutes, intrinsic immune cell signals, immunomodulation, wound healing, chronic wounds, skin tissue engineering, regenerative medicine

## Abstract

The immune system has a crucial role in skin wound healing and the application of specific cell-laden immunomodulating biomaterials emerged as a possible treatment option to drive skin tissue regeneration. Cell-laden tissue-engineered skin substitutes have the ability to activate immune pathways, even in the absence of other immune-stimulating signals. In particular, mesenchymal stem cells with their immunomodulatory properties can create a specific immune microenvironment to reduce inflammation, scarring, and support skin regeneration. This review presents an overview of current wound care techniques including skin tissue engineering and biomaterials as a novel and promising approach. We highlight the plasticity and different roles of immune cells, in particular macrophages during various stages of skin wound healing. These aspects are pivotal to promote the regeneration of nonhealing wounds such as ulcers in diabetic patients. We believe that a better understanding of the intrinsic immunomodulatory features of stem cells in implantable skin substitutes will lead to new translational opportunities. This, in turn, will improve skin tissue engineering and regenerative medicine applications.

## 1. Introduction

The skin is the largest organ in the human body performing essential functions, including body protection against the external environment, prevention of water loss from the body, temperature regulation, and ultraviolet (UV) absorption from sunlight to produce vitamin D [1,2]. Skin regeneration undergoes a dynamic and complex multistep process characterized by homeostasis, inflammation, proliferation, re-epithelization, and fibrosis. The wound healing is possible due to various platelets and immune skin cells secreted/activated by the number of cytokines [2,3,4,5,6].

Whereas minor superficial skin injuries usually heal by epithelialization alone, large and deep skin defects require a skin substitution to heal properly. Therefore, skin replacement therapies have evolved tremendously over the last few years [7]. Given the limited donor sites as it is in severely burned patients, tissue-engineered skin substitutes offer a promising treatment option for those cases [4,5,6,7,8,9,10,11,12,13,14,15,16,17].

In this review, we highlight the current progress in tissue-engineered skin substitutes used in skin replacement therapies. In particular, we focus on the immunomodulatory scaffolds and the role of immunomodulatory stem cells such as adipose and bone-marrow-derived mesenchymal stem cells used in cell-laden skin substitutes to repair skin.

## 2. Skin

Human skin is composed of three layers, the epidermis, dermis, and hypodermis with complex cells, nerve, and blood supply [17,18].

The epidermis is the outermost layer which is composed mostly of keratinocytes, arranging a stratified epithelium with basal keratinocytes at the innermost layer and the keratinized stratified squamous epithelium, which is known as the stratum corneum [19]. Moreover, melanocytes are present in the epidermal basal layer to form melanin being important for skin pigmentation and especially for the protection against harmful ultraviolet (UV) light [18]. Additionally, there are Langerhans cells present in the epidermis which act as a network of immune system sentinels. The main duty of these immune cells is to distinguish the appropriate adaptive immune response by interpreting the microenvironmental context in which they face foreign substances [20].

The dermis located below the epidermis is the thickest layer of skin. This layer is mainly composed of extracellular matrix (ECM) containing collagen type I, elastin, and glycosaminoglycans (GAGs), produced by fibroblasts [21]. The dermis has a major role in the biomechanics of the skin thereby providing mechanical strength and elasticity.

The hypodermis contains adipose tissue that is well vascularized and aids both the thermoregulatory and mechanical properties of the skin [18].

The stability and continuity of this complex multilayer organ can be disturbed and destroyed by wounds caused by different internal and physical or thermal external factors. A wound is a damage of the skin integrity and its function [22]. Wounds are usually classified based on the area of affected skin nature; the injured skin layers or the nature of the skin repair process [23,24]. Injuries that affect only the epidermal skin layer are called superficial wound, while injuries that damaged both the epidermis and dermal layers are referred to as partial-thickness wound. Full-thickness wounds are injuries of the epidermis, dermis including the sweat glands and hair follicles, and the underlying subcutaneous fat or deeper tissues as well [25].

Depending on the nature of the repair process, wounds are categorized into acute and chronic wounds. Acute wounds usually heal in an expected time frame (8–12 weeks) with minimal scarring [26]. Mechanical injuries which are sustained by abrasions and cuts (penetrating of knives or sharp objects) and surgical wounds are the primary sources of acute wounds. Moreover, burns and chemical injuries caused by radiation, corrosive chemicals, electricity, and thermal sources, are another class of acute wounds [26]. On the other hand, chronic wounds are tissue injuries that heal slowly and often show delayed healing as 12 weeks from the injury. Different factors such as diabetes, malignancies and ongoing immunosuppressive treatment, poor primary wound treatment, and persistent infections of wounds can impair the healing process, leading to chronic nonhealing wounds [25].

## 3. Skin Wound Healing Process

Skin wound healing is a complex dynamic and multistage process initiated by an injury. It requires the activation, recruitment or activity of numerous cell types such as keratinocytes, endothelial, fibroblast and inflammatory cells as well as growth factors, cytokines and chemokines [27]. In adults, the healing skin process is more restrictive than in children or embryo [27,28]. Fetal skin and skin of children have the intrinsic capacity for wound healing due to a rapid onset of this process following an injury. In contrast, delayed wound healing in the aged patients involves altered inflammatory response, such as delayed T cell infiltration into the wound bed and reduced cytokine production Additionally, re-epithelialization, collagen synthesis, and angiogenesis can be impaired in aged compared to young patients [29].

There are two main categories—primary and secondary wound healing. Primary wound healing requires straight, aseptic wound borders, which are close to each other. In contrast, during secondary wound healing, the wound borders are away from each other so that the wound has to be closed by granulation tissue [30].

**Figure 1:**
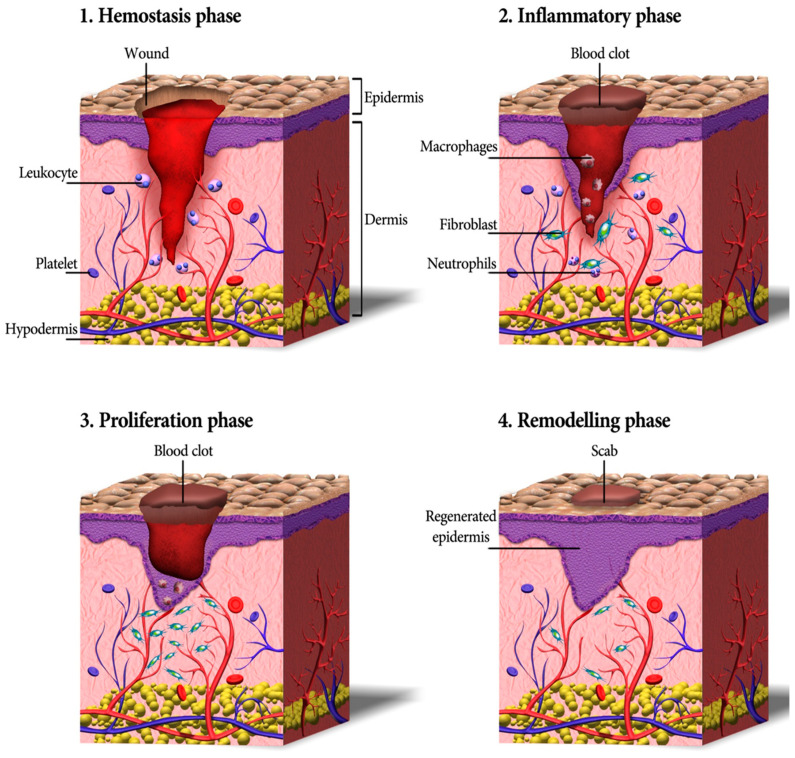
A schematic depicting the process of wound healing, including four continuous phases—homeostasis, inflammation, proliferation, and remodeling. Firstly, blood platelets are activated to form a blood clot and have a role in leukocyte recruitment. Next, neutrophils and macrophages clean the wound site from dead cells, bacteria, and other pathogens or debris. Then, fibroblasts migrate, proliferate, and activate the angiogenesis process. Finally, granulation tissue is formed, the deposition of extracellular matrix proteins occurs to reconstitute the dermal tissue, and the epidermis is regenerated. Eventually, many of the formed capillaries and fibroblasts disappear [17].

Cutaneous wound healing consists of a sequence of molecular and cellular events which occur after the onset of a tissue lesion in order to restore the damaged tissue. This process can be subdivided into four phases namely hemostasis, inflammation, proliferation, and remodeling (Figure 1) [17,31].

Hemostasis and Inflammation Phases

Most skin injuries, even superficial wounds, caused bleeding. Blood loss and the risk of infection is reduced by the clotting mechanism in which fibrinogen produced in the exudate stimulates a fibrin clot formation and seals the wound site. Therefore, hemostasis provides a protective barrier and contributes to successful wound healing [32]. The chemokines generated during hemostasis attract inflammatory cells and encourage resident immune cells. All those cells together start the inflammatory phase of wound healing within only minutes after the injury [33,34]. First, the vessels are dilated and the capillary permeability is enhanced. Then, local edema occurs and leukocytes such as macrophages and neutrophil granulocytes migrate into the wound. Cell debris is eliminated via phagocytosis to cleanse the skin wound [35]. The latter produces growth factors, cytokines, and other soluble mediators that activate keratinocytes, endothelial cells, fibroblasts, inflammatory cells, and other cells present in the wound. Neutrophils also produce antimicrobial peptides, reactive oxygen species (ROS), and proteases to kill and degrade potential pathogens [36]. It is worthy to mention that oxygen is critical in this step as ROS is generated in leukocytes by the nicotinamide adenine dinucleotide phosphate (NADPH) oxidase. Further, the formation of ROS has an active role in cytokine release [37].

The injury including damage of blood vessels triggers coagulation and is linked to the release of transforming growth factor-β (TGF-β), platelet-derived growth factor (PDGF) and VEGF. Those cytokines trigger tissue edema and inflammation. VEGF activates the migration of endothelial cells, leukocytes and epithelial cells to the wound [38]. Activated TGF-β provides rapid chemotaxis of neutrophils and monocytes to the wound site [38]. In addition, TGF-β induces leukocytes and fibroblasts to secrete TNF-α, IL-1, PDGF, and different pro-inflammatory chemokines. Pro-inflammatory chemokines are produced by cells primarily to recruit leukocytes to the site of injury [39].

As inflammation is settled, the wound advances into angiogenesis, matrix formation, and remodeling. Angiogenesis is triggered by mesenchymal stem cells secreting insulin-like growth factor 1 (IGF-1), PDGF-BB, VEGF, angiopoietin-1, and FGF.

Proliferation

Activated resident fibroblasts, endothelial cells, and keratinocytes are crucial cellular players facilitating the re-epithelialization and the formation of granulation tissue. These are two major processes occurring concomitantly during the proliferation phase [40].

First, the granulation tissue and blood vessels are formed by the in-growth of blood capillaries and lymphatic vessels into the wound site, viable endothelial cells at the wound borders start proliferation, and blood vessels start growing into the wound forming an organized microvascular network. Those processes are essential because the wound needs to be provided with oxygen and nutrients [41,42]. Then, a new vasculature is followed by the production of the extracellular matrix (ECM). Fibroblasts degrade the provisional matrix and synthesize new ECM in order to replace the injured tissue with a connective tissue scar. Thus, is mediated by different cytokines and growth factors starting with TGF-β that recruits fibroblasts synthesizing collagen I, III, and V, proteoglycans, and fibronectin [43]. Leukocytes are engaged in phagocytosis of debris and microbes and degradation of matrix. Recruitment of other blood cells as neutrophils and monocytes is enhanced by release of pro-inflammatory chemokines and cytokines, in particular TNF-α, IL-1, and IL-6. The newly synthesized ECM contains initially an enormous amount of hyaluronan that creates a structure enabling other migrating cells to penetrate into the wound area. When the granulation tissue is formed, some fibroblasts transform into myofibroblasts. This differentiation can be induced by TGF-β1. In addition to the presence of a soluble stimulus such as TGF-β1 found in inflammatory zone 1 (FIZZ1), other cytokines and mechanical stress are also needed to complete the differentiation [44]. Myofibroblasts generate a force that pulls the surrounding dermal and adipose tissue toward the wound. The formation of granulation tissue is a crucial part of wound healing [45,46]. An impaired granulation results in impaired wound healing and, in contrast, excessive granulation leads to a delayed re-epithelization [47,48]. On the other hand, epithelial cells respond to wounding by extensive changes in transcription, and the phenotype allows initiation of movement within a few hours after the injury [49].

Re-epithelialization is a critical phase of skin healing and it is characterized by replication and migration of epithelial cells across the skin edges in response to some growth factors such as FGF, epidermal growth factor (EGF), and keratinocyte growth factor (KGF). Moreover, matrix metalloproteinases (MMPs) such as MMP-1, 9, 10, and MMP-13 have been implicated in re-epithelialization. MMP-1 and MMP-9 promote human keratinocyte migration, the former tending to guide keratinocytes on fibrillar collagen in the basement membrane, allowing basal keratinocytes to detach and migrate toward the wound. MMP-10 is expressed by epidermal cells three days after wounding and it aids keratinocytes expression. MMP-13 also promotes re-epithelialization indirectly by affecting wound contraction and inducing keratinocyte migration [50,51]. As soon as the wound is covered with a monolayer of keratinocytes, their migration stops, and the formation of a (stratified) epidermis starts [52,53].

Remodeling (Maturation)

During the final phase of wound healing, remodeling, collagen is deposited in an organized and well-mannered network. Macrophages release some MMPs that trigger production of type VIII collagen, which is critical for local tissue integrity [34]. Net collagen synthesis will continue for at least 4 to 5 weeks after wounding. The increased rate of collagen synthesis during wound healing can be because of an increase in the number of fibroblasts and also from a net increase in the collagen production per cell [54]. Additionally, collagen type III is replaced by stronger collagen type I, which is followed by cleavage and cross-linking of fibrillar collagen [55]. In the remodeling stage, oxygen is required for mature collagen formation and fibroblast accumulation; it is necessary in the hydroxylation reaction of proline and lysine from procollagen chains in order to stabilize the triple helices of collagen [37].

The remodeling of the ECM components is the final and longest phase during the cutaneous wound healing. The granulation tissue matures forming a scar [56]. The ECM remodeling and the final scar formation can last up to 2 years after the wound is closed [25].

### 3.1. The Role of Macrophages and Pro-Inflammatory Cytokines in Wound Healing

Macrophages play critical roles in all phases of adult wound healing including inflammation, proliferation, and remodeling. Skin macrophages are derived from two different sources: a tissue-resident macrophage and circulating monocytes that are recruited to areas of injury and differentiate into macrophages. The first type includes a self-renewing pool of cells that originate from the embryonic yolk sack. These cells are named dermal macrophages which are permanent residents in healthy adult skin, and often found in nearby skin appendages. In contrast, during injury, bone marrow-derived monocytes are recruited to the skin injury, locally differentiate into macrophages and play key roles in wound healing. Both types of macrophages are involved in the wound healing process and they enable innate immune processes and play several crucial roles during wound healing [54]. Both types of macrophages are involved in the wound healing process and they enable innate immune processes and play several crucial roles during wound healing [55]. Additionally, macrophages secrete key pro-inflammatory and anti-inflammatory cytokines and therefore play a critical role in the regeneration and the wound healing phases. Traditionally, macrophages are classified into two cell phenotypes such as M1 and M2 depending on their cell surface markers and cytokine/chemokine production and function [56]. M1 macrophages are commonly associated with the pro-inflammatory process, whereas M2 macrophages are recognized as anti-inflammatory and proregenerative. It is also worthy to mention that distinguishing M1 and M2 phenotypes are only possible in vitro settings [54]. In in vivo environment, the existence of a heterogeneous subpopulation of macrophages with the characteristic of both M1 and M2 was reported [57,58,59]. The M1 phenotype macrophage is activated by pro-inflammatory signals including interferon-g (IFN- γ) and microbial products such as lipopolysaccharide (LPS). M1-macrophages can present high antigen and promote Th1 differentiation of lymphocytes which is producing pro-inflammatory cytokines in response to intracellular pathogens, leading to restriction of availability of microenvironmental iron to prevent bacterial infections [60,61]. M1 macrophages infiltrate the wound directly after injury and their number peaks at days 7–14 in the wound healing process. The activation of M1 is regulated by interferon-gamma (IFN-γ) as well as microbes. M1 triggers the production of pro-inflammatory cytokines such as TNF-α, IL-6, IL-12, and CC chemokine ligand 2 (CCL2) [62].

**Figure 2:**
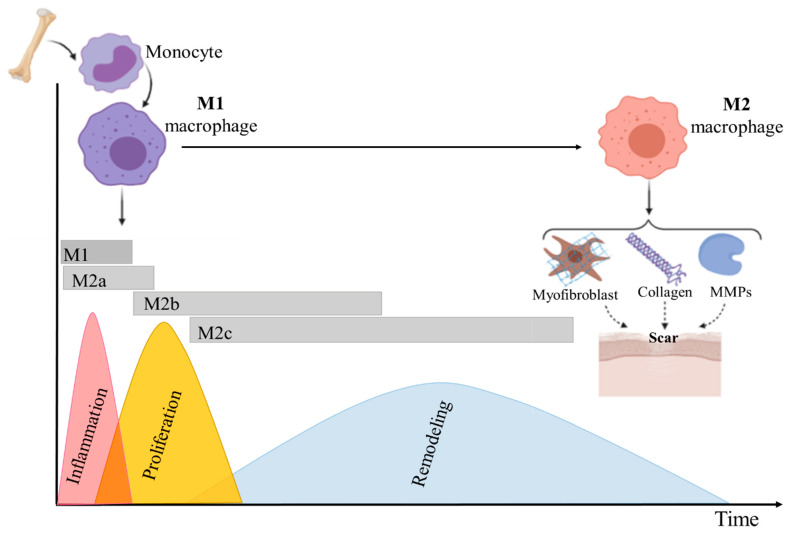
Schematic representing macrophage plasticity in wound healing. As the wound healing stages progress and the wound microenvironment changes, M1 pro-inflammatory macrophages undergo a phenotypic switch to an M2 anti-inflammatory and proregenerative state, which stimulates tissue regeneration. Thus, macrophages regulate the transition from the inflammatory to the proliferative phase of healing. Furthermore, macrophage phenotypic switch from M1 to M2 effects scar formation by inducing fibroblast proliferation, myofibroblast differentiation, synthesis of different MMPs, and various types of collagen.

The second type of macrophages, alternatively-activated M2 cells, plays a role in the late process of wound healing and scar formation. M2 can be induced by IL-4 or/and IL-13, and their population increases at 14–28 days after wounding [63]. The secretion of anti-inflammatory factors such as IL-10, TGF-β1, home oxygenase-1 (HO-1), and arginase characterized M2 macrophages [64]. The role of M2 macrophages is also induction of fibroblast proliferation, myofibroblast differentiation, and synthesis of different MMPs and various types of collagen, mainly VIII and I collagen (Figure 2). M2 can be further subdivided into different subgroups: M2a, M2b, and M2c, based upon the inducing agent and molecular marker expression. The induction of M2a-macrophages is triggered by IL-4 and IL-13, while M2b-macrophages are activated by immune complexes and toll-like receptors (TLR) agonists and M2c can be activated by glucocorticoids and IL-10. During the wound healing process, M2a acts as an anti-inflammatory agent and aids wound healing. M2b macrophages regulate the breadth and depth of the immune response and the inflammatory reaction [65,66,67]. M2b macrophages can express and secrete substantial amounts of the anti-inflammatory cytokine IL-10 and low levels of IL-12, which is the functional converse of M1 cells; the main roles of M2c are in immunosuppression, phagocytosis, and tissue remodeling [68,69].

According to some reports, macrophages phenotype (subpopulation) depends mostly on the time of monocytes recruitment [56]. Other studies suggested that monocytes phenotypes are determined by stimuli in the wound healing environment. These stimuli change cell polarization and result in the differentiation of M1 macrophages into M2 macrophages (Figure 2) [67].

#### Pro-Inflammatory Cytokines and Their Role in the Wound Healing

Pro-inflammatory cytokines primarily trigger recruiting of leukocytes to the sites of injury. Another important role of pro-inflammatory cytokines is in the activation and orchestra ratio of the specific signal pathway and silent genes under the cellular healing process [68]. TNF-α, IL-6, and IL-1 are the key pro-inflammatory cytokines that are involved in the wound healing process [69]. TNF-α controls inflammation, protects tissue from infection, and triggers the synthesis of other cytokines in wound healing. Another multifunctional cytokine is IL-6 that activates several important pathways during skin wound healing. IL-6 is responsible for the skin fibroblast activation which, in turn, promotes cell migration into the wound and the expression of progenitor factors. The main role of IL-1 is the activation of a cascade characteristic of innate immunity toll-like receptors [70]. Although the physiological function of pro-inflammatory cytokines has been studied, the knowledge on their specific role in skin regeneration is still limited and requires further investigation [68].

Macrophages and their production/activation of pro-inflammatory cytokines play an important role in chronic nonhealing skin wounds where the wounds remain in the inflammation phase and cannot heal [71]. A chronically elevated expression of pro-inflammatory cytokines such as TNF-α, IL-1, IL-6, and MMPs may occur in patients with the systemic autoimmune and metabolic disease [69,72,73].

Prolonged inflammation and failure of proper transit from the regenerative to the resolving phase of the healing process may cause excessive scarring and/or overgrowth of granulation tissue, called a human keloid scar [74,75,76]. The studies showed a higher infiltration with M2 macrophages than with M1 macrophages in a keloid [75,76].

It is believed that tracking the macrophages population, decreasing the number of macrophages, and/or changing their polarization could affect the wound healing process and scar formation [77]. Thus, this change may help to develop therapies for improving healing and decreasing scarring [78].

### 3.2. The Role of Growth Factors in Wound Healing

Growth factors are polypeptides that regulate the growth, differentiation, and metabolism of cells, and they control the biological events of different wound healing phases. Growth factors are upregulated in response to tissue damage and are secreted by platelets, leukocytes, fibroblasts, and epithelial cells. They can bind to specific cell surface receptors to act through autocrine, paracrine, or endocrine mechanisms and control the process of tissue repair [68,79]. Binding to receptors results in a cascade of events that activate the cellular machinery to facilitate wound healing [80]. In the first stage of wound healing, hemostasis, the clot, and surrounding wound tissue release pro-inflammatory cytokines and growth factors such as TGF-β, PDGF, FGF, and EGF. For example, in a research study, it was shown that TGF-β level increased initially after wounding and then declined gradually with wound closure [79]. Afterward, during the inflammation phase, dendritic epidermal T cells (DETC) are activated by damaged, stressed, or transformed keratinocytes and produce fibroblast growth factor 7 (FGF-7), KGF, and IGF-1, to support keratinocyte proliferation and cell survival. Moreover, angiogenesis, which is necessary for normal healing, occurs during the proliferative stage. The vascularization in wound healing process can be triggered by some growth factors including IGF-1, PDGF-BB, VEGF, and FGF2. In addition, the growth factor TGF-β1 has a key function in the proliferation phase of wound healing. TGF-β1 induces the migration and proliferation of fibroblasts, which can improve granulation tissue formation, collagen synthesis, and angiogenesis promotion. Moreover, the hepatocyte growth factor (HGF) is another growth factor that is mainly produced by fibroblasts and has an interaction with cytokines in wound healing. HGF and its tyrosine kinase receptor, c-Met (mesenchymal epithelial transition factor), are expressed on the surface of keratinocytes. HGF promotes important steps in granulation tissue formation and neoangiogenesis. It was shown that during the wound healing process, suppression of HGF in mice results in delayed granulation tissue formation and decreased neovascularization, and HGF can suppress VEGF-mediated inflammation [81,82]. Furthermore, growth factors from the EGF family, such as EGF, heparin-binding EGF (HB-EGF), and TGF-α produced by macrophages and keratinocytes, exert important effects in covering wound surfaces with epithelium. These autocrine ligands interact with EGF receptors on keratinocytes and induce downstream mechanisms fostering keratinocyte proliferation and migration, and consequently re-epithelialization. In the last healing stage, remodeling, TGF-β induces the expression of MMP-9, which is important for matrix remodeling and angiogenesis.

## 4. Conventional Skin Wound Treatment Options

Different treatment approaches have been established to improve the healing of various types of wounds. Skin grafting is one of the most common options for wound treatment by using autografts, allografts, or xenografts.

A split-thickness skin graft, also known as a partial-thickness skin graft, contains the epidermis and varying portions of the dermis. Split-thickness grafts are shaved from the patients’ own skin. It can be processed through skin meshes which makes apertures onto the graft, allowing it to expand up to nine times its size, and further placed on the wound site [83]. Although the application of autografts (graft from the same individual) decreases immunological rejection of tissues, there might be the donor site shortage, as in heavily burned patients [84]. In contrast, allografts (graft from the same species but different individual) can solve this limitation and be used as temporary skin coverage. When used in a viable cryopreserved form, allografts contain blood capillaries, which can rapidly connect to the underlying host vessels at the wound site and restore blood supply over the first 3 days after the application and full circulation after 4–7 days. Therefore, allografts remain a standard temporary wound cover [85]. Nevertheless, problems linked to human allografts are the high risk of immune rejection, infection potential, and problems of variability in the quality of the tissue [86].

Xenograft or heterograft is a skin graft transplanted from one species to another. As such, porcine-derived xenografts are most frequently used, as they are convenient and easily available. Split-thickness porcine xenografts are harvested and either preserved in glycerol or cryopreserved to provide a readily accessible wound dressing [87,88]. However, the high risk of immune rejection to xenografts and even in allografts in some cases and transmission of pathogens remain the main concerns. In particular, skin xenografts are prone to cellular rejection, which is similar to the mechanism detected in allografts [89].

These various limitations of the abovementioned grafts and grafting procedures inspired their development and eventually the clinical application of some decellularized dermis products, such as alloderm and allopatch and other bioengineered skin substitutes [90]. Different types of engineered skin substitutes have been developed to regenerate skin by mimicking its composition, texture, and function. Tissue engineered skin substitute contains different skin cells and/or ECM [91]. Thus, bioengineered skin substitutes can provide both epidermal and dermal components required to obtain an efficient full-thickness wound closure and regeneration [1]. The presence of an efficient number of cells, particularly stem cells, in appropriate tissue engineered skin substitutes allows regeneration of native-like skin in wounds by promoting cell migration, differentiation, and vascularization [92].

## 5. Application of Stem Cells in Skin Substitutes

According to the findings obtained by McCulloch and Till, based on [93,94], stem cells can be defined two prominent features: (1) they are undifferentiated and renew themselves for the entire life span and (2) they have an extraordinary potential to develop from a common precursor into multiple cell types with particular functions [15]. Stem cell-based therapies have the potential to enhance cutaneous regeneration due to their ability to secrete proregenerative cytokines modulating immune response, making them an appreciated option for the treatment of chronic wounds [95]. However, stem cell therapies are limited by the need for invasive harvesting techniques, immunogenicity, and limited cell survival in vivo [96]. Embryonic stem cells (ESCs), induced pluripotent stem cells (iPSCs), and adult stem cells are among the main sources of cells that have been used in various experimental research for wound treatment and regeneration of injured skin [97]. In this review, we discuss mainly the characteristics and applications of iPSCs and, in particular, mesenchymal stem cells (MSCs) including bone marrow-derived mesenchymal stem/stromal cells (BMSCs) and adipose-derived stromal/stem cells (ADSCs) in skin substitutes, along with the immunomodulatory effects of MSCs.

### 5.1. Induced Pluripotent Stem Cells

iPSCs are able to reproduce all types of adult cells in the course of their differentiation and they have an unlimited self-renewal capacity [98]. Before studying the iPSCs, ESCs were the only well-studied source of pluripotent stem cells. ESCs can be obtained from the inner cell mass and/or epiblast of blastocysts [99]. Although there are some protocols for the preparation of various cell derivatives from human ESCs, there are restrictions for ESC use in cell replacement therapy, such as the incompatibility between the donor cells and the recipient, which can result in the rejection of transplanted cells [100]. Then, iPSCs were obtained later by reprogramming animals [101,102,103,104] and human differentiated cells by induced expression of transcription factors including Oct4/Sox2/c-Myc/KLF4 or Oct4/Sox2/NANOG/LIN28 [105,106]. iPSCs closely resemble ESCs in a broad spectrum of properties, closely such as cell morphology and proliferation, sensitivity to growth factors, and signaling molecules. Moreover, similar to ESCs, iPSCs are able to differentiate in vitro into derivatives of all three primary germ layers (ectoderm, mesoderm, and endoderm) and form teratomas following their subcutaneous injection into immunodeficient mice [100,105,106]. Therefore, iPSCs can be an appropriate alternative for ESCs in the area of clinical application of cell replacement therapy.

iPSCs are derived from adult cells by in vitro induction of pluripotency with noninvasively harvesting, and can be transplanted autologous, reducing immune rejection. Importantly, iPSCs are the only cell type capable of being differentiated into all cell types of normal skin, and therefore they have been widely utilized in wound healing applications. Owing to their high differentiation capacity into descendants of all three germ layers, iPSC-derived cells have the potential to enhance each of the phases of diabetic wound healing through their paracrine and direct cellular effects [96]. During the inflammatory phase, iPSC-derived cells secrete growth factors and cytokines, counteracting the suppressed cytokine secretion profile seen in diabetic patients [107,108]. This process eventuates in the recruitment of macrophages and proliferative cells including fibroblasts and keratinocytes, which are known to be deficient in chronic wounds [109,110,111,112]. Direct application of stem cells into the wound bed also mitigates the impaired homing potential of progenitor cells into diabetic wounds [113]. In the proliferative phase, different cells including endothelial, fibroblasts, pericytes, smooth muscle, keratinocytes, or MSCs are derived from potential iPSC [96,114,115], afterward increasing angiogenesis and promoting collagen deposition [110,111]. Since the remodeling phase is extremely dependent on functional myofibroblasts, their recruitment during the proliferative phase is vital to the last stage of wound healing. Eventually, iPSCs retain the ability to differentiate into keratinocytes [114]. In a review by Gorecka et al. [96], the potential and limitations of iPSCs in wound healing applications were fully examined. In this regard, Table 1 demonstrates major findings of studies relating to wound healing in a murine model [96].

### 5.2. Bone Marrow-Derived Mesenchymal Stem/Stromal Cells

Bone marrow is an important compartment of bone regulating its homeostasis. Bone marrow itself can be considered as an immune organ containing distinct cell types, which secrete a large number of cytokines and growth factors that can have angiogenic, anti-inflammatory, anti-apoptotic and immunomodulatory effects [121,122]. Moreover, bone marrow is a metabolic organ and has been demonstrated to regulate whole-body energy metabolism. Thus, the cellular composition of bone marrow can change with age, gender, and metabolic activity [122,123]. The adult bone marrow is composed of hematopoietic cells and the associated supporting stroma. The stroma consists of cells with multipotent differentiation capacities that are usually considered mesenchymal stem cells [124].

**Figure 3:**
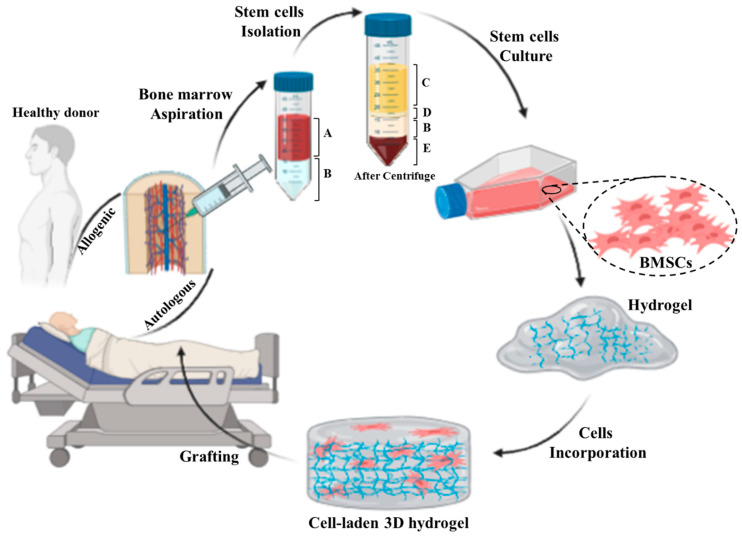
Schematic representing preparation process of an autologous/allogenic stem cell-laden hydrogel for skin regenerative approaches: the bone marrow is aspirated from donor or patient own and then the stem cells are isolated from bone marrow. Afterward, the BMSCs are cultured and incorporated/encapsulated in a hydrogel solution. Finally, the cell-laden hydrogel is pre/post-crosslinked and grafted to the injured site. (A: whole blood, B: Ficoll gradient, C: plasma, D: mononuclear cell, and E: red blood cells).

Bone marrow-derived mesenchymal stromal cells (BMSCs) are multipotent stem cells capable to differentiate into numerous cell types, including fibroblasts, endothelial cells, cartilage, bone, muscle, and neuronal cells. BMSCs also secrete a large number of growth factors and cytokines that are critical for the repair of injured tissues [125,126]. Because BMSCs are able to differentiate into multiple cell types and produce tissue repair factors, BMSCs skin substitutes provide an alternative to conventional treatments for skin repair [127]. In numerous animal and human studies, BMSCs were directly injected into skin wounds [128,129,130,131]. The results indicate that both autologous and allogeneic BMSCs could induce tissue regeneration and accelerate wound closure. However, the direct injection of cells showed only a small therapeutic efficacy with side effects such as invasive procedure with attendant risks, and accumulation of cells for a long time [132,133,134]. Therefore, the integration of stem cells into engineered scaffolds such as hydrogel networks offers controllable mechanical, physical, and chemical properties, which can improve the integration of a skin substitute into the host tissue (Figure 3). Thus, the integration of BMSCs into an appropriate scaffold can sustainably support the healing process by creating a proregenerative microenvironment in the wound area [135].

**Figure 4:**
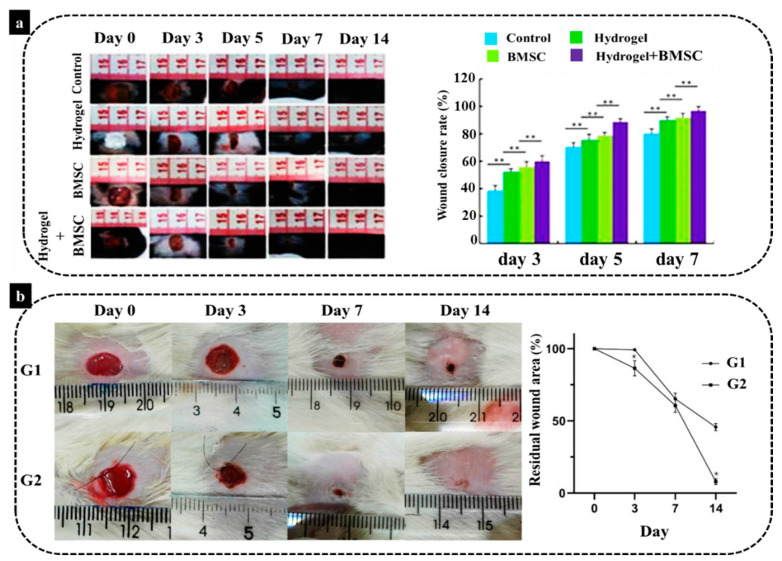
(**a**) Wound healing conditions of mice in the control group, the hydrogel group, the BMSCs group and the hydrogel–BMSCs combination group, 3, 5, 7, and 14 days after the operation with comparison of the wound healing rates of mice (** *p* < 0.01). (**b**) The comparison of wound area in two different groups (G1: without treatment and G2: treated with BMSCs plus the biomaterial, * *p* < 0.05) and the graph of residual wound site [136,137].

In an animal study by Lei et al. [136] a novel thermosensitive NIPAM (N-Isopropylacrylamide) hydrogel was employed to provide substrates for transplanting BMSCs for the management of severe skin wound healing. Their results demonstrated the injection of BMSCs on the wound site could aid wound closure and tissue regeneration. However, the delivery of BMSCs by hydrogel combination exhibited a noticeably more effective therapeutic effect than wound treated with BMSCs alone (Figure 4a). This hydrogel-BMSCs combination therapy improved fibroblast proliferation in the dermis, as fibroblasts started to deposit ECM, in particular collagen which plays the main role in skin wound repair. Moreover, another study carried out by Viezzer et al. [137] showed an improvement of wound healing of ulcers in a diabetic rat model. The authors transplanted chitosan-based polyurethane hydrogels containing rat-derived BMSCs with a continuous degradation in an aqueous solution. Their findings illustrated that the animals treated with the BMSCs hydrogels had a significantly better regeneration rate than their counterparts from the control group (without treatment) after 14 days, with a significant reduction in wound size (Figure 4b). The authors highlighted in their study the anti-inflammatory role of BMSCs in healing of chronic wounds. This is an essential aspect of foot diabetic ulcers that have a prolonged inflammatory phase with a pro-inflammatory profile leading to a chronic form of the wound. This prolonged inflammation is related to the prolonged healing of these wounds, for example, due to recurrent infections. Diabetic ulcers may lead to extremity amputation and even death. Application of BMSCs was shown to significantly reduce inflammation and improved the neovascularization around the nonhealing wound. Therefore, it can be concluded that BMSCs loaded in an appropriate hydrogel scaffold could provide a suitable wound dressing for diabetic patients, and it can be also used for various wound healing approaches.

To develop stem cell-based scaffolds, stem cells need to be first harvested from animals or humans and then loaded into the prepared scaffold. Hence, to avoid repeated harvesting of BMSCs for each treatment and cell loading into scaffolds, cryopreservation is vitally important [138,139]. Recently, an animal study was conducted to compare the efficiency of freshly isolated and cryopreserved BMSCs on wound healing [138]. For this, both fresh and 30-day preserved BMSCs were cultured on monofilament polypropylene scaffolds and used for therapeutic purposes in guinea pigs. There was no significant difference in population doubling time between fresh and cryopreserved BMSCs and both of them expressed cell surface markers (CD73, CD90, and CD105) and mRNA without significant difference. Additionally, both pre- and post-thaw BMSCs were successfully differentiated into three different cell lineages including chondrogenic, osteogenic, and adipogenic lineages. Moreover, a significant difference was detected in wound contraction between cell-treated wounds and control groups, while there was no difference observed among scaffold-augmented MSCs, both pre- and post-thaw, and the MSCs-only group.

To summarize, BMSCs are capable to differentiate into various cell types and secrete numerous bioactive factors that improve granulation tissue formation, angiogenesis, and decrease inflammation. Furthermore, they can be easily cryopreserved to be used for later applications. Therefore, BMSCs-based skin substitutes represent one of the promising approaches to heal hard-to-heal wounds.

### 5.3. Adipose-Derived Stromal/Stem Cells

Recently, adipose tissue has been highlighted as a promising source in the field of cell therapy and regenerative medicine. Adipose tissue is composed of multiple cell types such as mature adipocytes and the stromal vascular fraction (SVF), which is a source of adipose-derived stromal/stem cells (ASCs), endothelial cells, pre-adipocytes, lymphocytes, and adipose-resident macrophages [4,140].

In contrast to bone marrow, adipose tissue represents advantages in terms of its accessibility and abundance for use in tissue engineering and regenerative medical applications [16,141]. The potential utility of ASCs has been demonstrated in multiple preclinical animal models [142,143]. Similar to BMSCs, the ASCs have been characterized based on their immunophenotypic and differentiation properties [144,145,146,147]. Because large volumes of adipose tissue can be easily obtained from individual donors, it is possible to obtain a high yield of 375 ± 147 × 10^3^ cells per mL of lipoaspirate [146]. The clinical application of adipose-derived cells has developed considerably in the past two decades. Both the heterogeneous adipose stromal vascular fraction (SVF) and the more homogeneous adipose stromal cells (ASCs) offer distinctive opportunities as novel cell-based treatments for tissue regeneration [148]. Additionally, adipose-derived cells have shown great potential in various therapeutic fields such as osteoarthritis, scleroderma, multiple sclerosis, renal insufficiency, erectile dysfunction, and wound healing [148,149].

ASCs are mesenchymal cells that have a capacity for self-renewal and can be differentiated into chondrocytes, adipocytes, myocytes, neurocytes, and osteoblasts, among other cell lineages. Therefore, ASCs have been widely employed in clinical trials, for example, for the treatment of diabetes and chronic wounds [150]. Generally, therapeutic effects of ASCs are mainly due to their high differentiation capacity, secretion of pro-healing growth factors and cytokines, and active remodeling of the extracellular matrix [151]. Lee et al. [152] and Bura et al. [153] explored the effects of cultured ASCs administrated intramuscularly on revascularization of critical limb ischemia. In the study of Lee et al. [152], ulcers were healed successfully in 66.7% of twelve patients after six months, with only some mild complications including transition mild fever, flu-like syndrome, pain, and headache. In addition, pain reduction and improved walking distance were detected after six months. In the trial of Bura et al. [153] in three of seven patients, the suffering of nonrevascularizable critical limb ischemia, limb amputation was prevented and they also showed decreased pain and increased tissue oxygen pressure compared to before ASCs injection.

Unfortunately, both the abovementioned studies employed only a small sample size and therefore represent a low level of evidence due to the lack of controls and randomization. However, in those two studies, it was undoubtedly the application of ASCs that improved ulcer healing due to enhanced angiogenesis. However, some patients were still unresponsive to the treatment. This might be partially explained by the impaired migration of injected ASCs from the muscle to the target tissue, along with their reduced differentiation capacity and paracrine effects after their in vitro cultivation [154].

More recently, the SVF, which contains both endothelial cells (ECs) and ASCs with a multitude of regenerative properties, has been used in different wound healing applications [4,11]. SVF cells have a high intrinsic angiogenic potential due to the secretion of multiple proangiogenic factors [155]. Moreover, the stem cell content and the proliferation capacity of SVF cells are not heavily dependent on donor age [156,157]. This aspect is essential regarding their possible regenerative applications.

**Figure 5:**
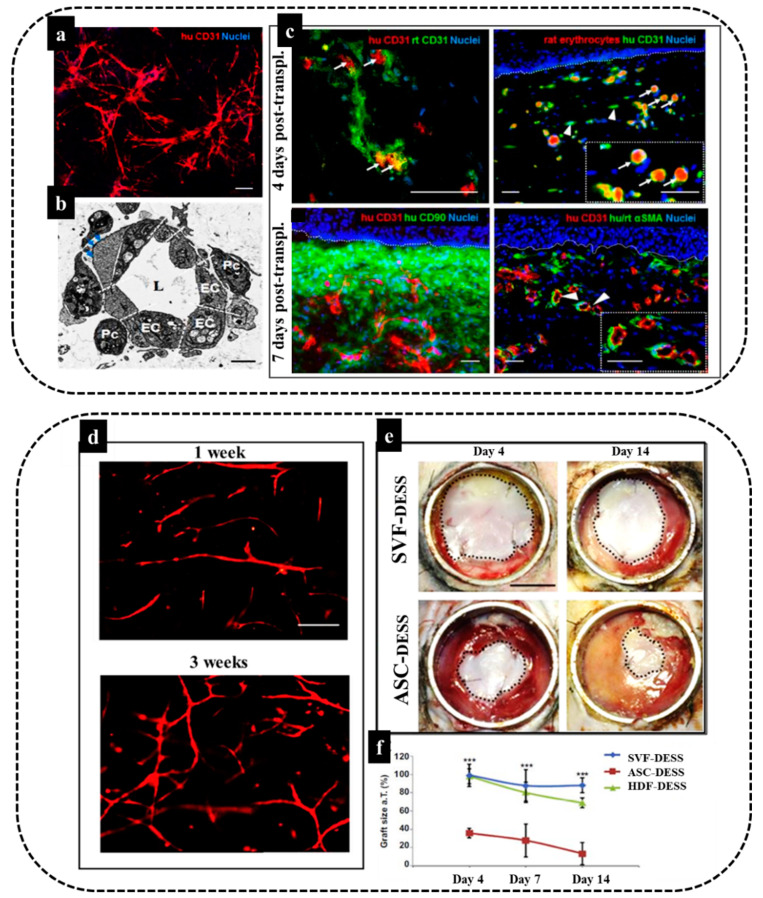
Engineering of dermo-epidermal skin substitutes with adipose-SVF cells. (**a**,**b**) Endothelial cells derived from freshly isolated SVF and perivascular cells in fibrin–collagen type I hydrogel demonstrate in vitro tubular-like structure formation and in vivo anastomosis with the host vasculature. Formation of a complex network of interconnected capillaries after 21 days in culture. Human bioengineered capillaries are stained for human-specific CD31 marker (red) and cell nuclei with Hoechst (blue) and transmission electron microscopy showing a cross-section of an in vitro grown capillary. Note the presence of a central lumen (L), which is surrounded by multiple ECs (EC) covered by pericytes (Pc). The deposition of basement membrane (BM) (blue arrows) was also detected. (**c**) Establishment of a functional connection (white arrows) between human CD31-positive capillaries (red) and rat CD31-positive capillaries (green) already 4 days post-transplantation. This connection was further confirmed by the presence of rat erythrocytes (red autofluorescence) in the lumina of human CD31-positive capillaries (green) (white arrows). The inset shows a magnification of the area indicated by white arrows. White arrowheads indicate nonperfused human capillaries. Moreover, representative section of a highly vascularized human dermo-epidermal skin substitute after 7 days post-transplantation is demonstrated. The engineered capillaries are visualized by the human specific CD31 antibody costained by human CD90 marker delineating the human dermal compartment. Staining for human/rat aSMA (pericyte marker) reveals that the majority of transplanted capillaries were already covered by pericytes in vivo. Hoechst stains the nuclei blue. White dotted lines indicate the dermo-epidermal junction [8]. (**d**) Optimization of vascular network formation in vitro to determine the optimal culture time for maximal in vitro capillary network formation, fibrin hydrogels containing SVF stained using a human specific CD31 antibody at one and three weeks of culture, (**e**) The SVF–DESS capillary plexus reduces shrinkage and accelerates the establishment of tissue homeostasis. Black dotted lines indicate the area of each skin transplant used for planimetry analysis and (**f**) the skin graft coverage area in was significantly improved in SVF–DESS as compared to control groups (*** *p* < 0.001) [4].

In this respect, we confirmed vasculogenic properties of the SVF by phenotypic and functional characterization of the freshly isolated CD31^+^/CD34^+^ ECs and CD31+CD34- ASCs [8,11,158]. These two cell populations present in the SVF developed spontaneously into mature, highly branched, and interconnected vascular networks when cultured in a 3D scaffold [8]. In a particular study, we explored the use of human endothelial cells derived from freshly isolated adipose stromal vascular fraction (SVF) in a 3D coculture model of vascularized skin substitute in full-thickness wounds in immune-deficient rats [8]. Results demonstrated the rapid graft–host vessel anastomoses and blood perfusion (Figure 5a–c). Moreover, we successfully applied freshly isolated SVF cells to generate a prevascularized human dermo-epidermal skin substitute (DESS) based on a 3D hydrogel coculture system [4]. As demonstrated in our studies, these capillary networks showed a rapid inosculation in an animal model and thus improved the wound healing process of the skin substitutes [4]. In particular, we employed fibrin hydrogels as a 3D matrix and an optimal number of SVF cells were seeded in the hydrogel to generate a functional and homogeneous dermal capillary plexus prior to transplantation (Figure 5d). Fibrin is the physiological matrix present at the onset of wound healing and acts as an important adhesion site for leukocytes and endothelial cells during tissue regeneration. Moreover, the stiffness, biodegradability, and pore size of fibrin hydrogels might strongly influence and direct the lineage-specific cell differentiation and secretome of loaded stem cells [159,160,161]. Thus, fibrin hydrogels can provide an appropriate matrix to encapsulate SVF to prepare a DESS. The in vivo analysis after implantation of SVF based DESS (Figure 5e) showed the graft size of the SVF-treated group was significantly increased at day 4, day 7, and day 14, as compared to the ASC only-based transplants.

These results demonstrate that SVF-based prevascularization strongly supports epidermis formation and maturation, similar to human dermal fibroblasts at days 4 and 7 following transplantation, and it could also reduce graft contraction (Figure 5f).

To conclude, submerged within an appropriate 3D environment, SVF cells allow for efficient in vitro prevascularization of human autologous dermo-epidermal skin grafts. This confirms their possible future clinical application [4].

**Figure 6:**
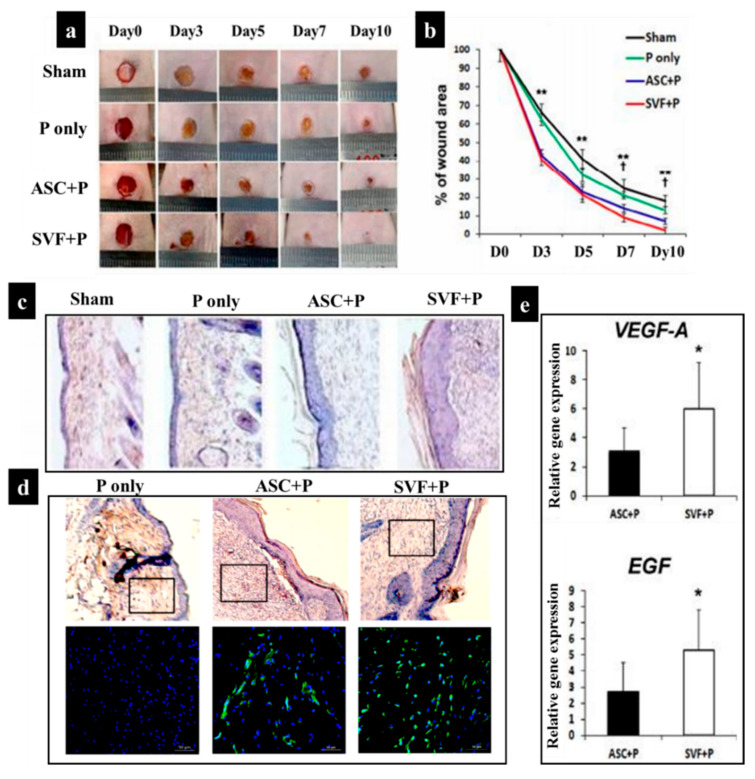
(**a**) Wound contraction over a 10-day period in four groups: sham, pluronic hydrogel (P) only, ASC+P, and SVF+P; (**b**) diagram showing the percentage of wound area each day; (**c**) histological images after H&E staining at 14 days after the injection of cells. (**d**) Representative photographs of isolectin B4 (ILB4), which is a marker of endothelial cells for neovascularization, in the skin wounds at 14 days after cell transplantation; nuclear DAPI staining is blue and ILB4 staining is green. On the upper line of the picture are the results of H&E from wound tissue after cell injection. The black rectangular box indicates the location of endothelial cells that show capillary density. (**e**) Quantification of expression levels for VEGF-A and EGF in skin wound tissues (* *p* < 0.05, ** *p* < 0.01) [162].

Additionally, some other studies have demonstrated the application of SVF-loaded scaffolds for wound healing. For example, in the study of Chae et al. [162], a pluronic hydrogel was employed to deliver human ASCs and SVF into the wound site via injection. The comparison of four different categories including sham, pluronic hydrogel only (P), pluronic hydrogel with ASCs (ASC+P), and a pluronic hydrogel with SVF (SVF+P) in vivo demonstrated that SVF+P injection into the dermis triggered the strongest wound contraction, improved cellularity, and re-epithelialization process in comparison with sham, P, and ASC+P (Figure 6). This effect was mainly due to the high vasculogenic potential of SVF and high secretion of vascular endothelial growth factor (VEGF-A) and epidermal growth factor (EGF) (Figure 6). Notably, EGF plays an essential role in epithelialization by promoting the migration and proliferation of keratinocytes and fibroblasts via the phosphoinositide 3–kinase signal and extracellular signal-regulated kinase pathways [163,164,165].

Moreover, in a clinical study by Nilforoushzadeh et al. [166], a skin graft containing human SVF cells encapsulated in a fibrin-collagen hydrogel was used to enhance the wound healing in diabetic patients. The results showed, for the first time, that SVF-based full-thickness skin grafts were safe and accelerate the wound healing process when compared to commercially available dermo-epidermal skin grafts. This study confirms the significant advantage of using SVF for prevascularization of skin grafts for the treatment of difficult-to-heal wounds [4].

Furthermore, a study by Guo et al. [167] has proved that human-derived ASCs delivery in a biomimetic-collagen scaffold can accelerate diabetic wound healing in a similar fashion as BMSCs. They illustrate that wounds of diabetic mice treated with ASCs or BMSCs could contract and wounds healed in the same manner. Importantly, ASCs or BMSCs treated wounds healed more rapidly than acellular-treated wounds. However, ASCs represent a more attractive cell source in comparison with BMSCs because ASCs require less painful and invasive isolation procedures, are very stable under in vitro culture conditions, demonstrate a rapid expansion in vitro, and contain a 40-fold higher number of stem cells than the BMSCs [168,169].

## 6. Immunomodulatory Properties of MSCs: ASCs and BMSCs

Clinically, large dermal wounds require skin replacement to protect exposed tissue and induce tissue regeneration. However, existing therapies frequently leave these patients with painful and disfiguring scars. Severe scars can significantly impair growth and movement and often require several follow-up surgeries. Importantly, the dermal ECM components such as collagen I, III, fibronectin, and elastin present in skin substitutes can modulate scarring.

Since the immune system was demonstrated to play a critical role in modulating scar formation, recent research focused on the support of immune cells to facilitate scarless wound healing.

It is known that MSCs can modulate the immune system and regulate skin tissue regeneration. Importantly, MSCs can mitogen- and allo-activated lymphocyte proliferation [170,171]. This effect is heavily dependent on some factors; for example, MSCs inhibit lymphocyte proliferation mainly via the secretion of TGF-β1, IL-10, HLA-G, nitric oxide, and hepatocyte growth factor, as well as due to the expression of indoleamine 2,3-dioxygenase (IDO enzyme) [172,173,174]. Further, MSCs secrete trophic factors which are critical for vasculogenesis and angiogenesis, and aid tissue regeneration [175,176]. Additionally, MSCs have been administrated to the site of wound in animal models by encapsulation in gelatin microspheres and microcryogels or loading into a 3D graphene foam [177]. It has been shown that 3D graphene foam loaded with MSCs released prostaglandin E2 (PGE2), which suppresses the release of pro-inflammatory cytokines including TNF-α, IFN-γ, IL-6, IL-8, and IL-12p70, and increases the release of anti-inflammatory cytokines such as IL-10 and IL-12p40, and TGF-β1 by macrophages [178,179,180]. Additionally, PGE2 reduced the proliferation of T cells in the wound and is a cofactor in the transition from TH1 to TH2 cells, which decrease tissue inflammation and, further, tissue regeneration [179]. Moreover, higher levels of IL-10 expressed by T cells and macrophages in response to PGE2 can limit or reduce the inflammatory mechanism of immune cells. IL-10, which is an important anti-inflammatory cytokine, inhibits further neutrophil invasion and respiratory burst [180]. IL-10 also affects fibrosis by downregulating the release of TGF-β1 in T cells and macrophages, and remodeling ECM by reprogramming wound fibroblasts. IL-10 has direct effects on the prevention of excessive collagen deposition by reducing the expression of pro-inflammatory cytokines such as IL-6 and IL-8 in the wound environment [181]. Finally, IL-10 expression results in resolution of inflammatory stage and rushing of the wound into the proliferation stage and over-expression of IL-10 can produce an environment in which wound healing tends to occur without scar formation [182,183,184].

Therefore, allogeneic MSCs have been utilized for treatment of different diseases, especially for scarless skin regeneration. MSCs from different sources are similar in a range of phenotypic and functional features. However, there are subtle differences that may result from the microenvironmental niche, the local, and the ontogenetic age or induced by the isolation and culture procedure [185]. Therefore, the immunomodulatory properties of BMSCs and ASCs, in particular due to their paracrine effects, have also been characterized in detail [186,187,188,189].

ASCs represent an interesting source of MSCs, which can be easily obtained and used for autologous applications as they reduce apoptosis and improve tissue repair and angiogenesis and affect immunoregulation [190,191]. In general, there are several considerations regarding the immunostimulatory or immunosuppressive effects of ASCs, including (1) the incapability of passaged ASCs to excite an allogeneic immune reaction [192], (2) the effect of soluble factors and cell-to-cell contact in stimulating an immunosuppressive response [193,194,195], and (3) the potential for tumor development or growth [196,197,198]. Thus, based on different studies one hypothesis is that early passages of ASCs express markers, such as MHC II, CD45, CD80, and CD86, which trigger antigen presenting cells (APCs) and immune response [192,196]. Nevertheless, these APC-associated markers are lost by continued passaging of ASCs, reducing the immune response until it has been removed. Therefore, the immunophenotypic changes of ASCs are directly associated with their ability to perform as stimulator cells. In another hypothesis, the T cell response is activated and the expression of some factors including IDO, PGE2, hepatocyte growth factor, and leukemia inhibitory factor is reduced [193]. IDO is an enzyme that catalyzes the rate limiting step in the conversion of L-Tryptophan to NAD+ via the de novo pathway. The secreted active bioactive factors downstream of IDO upregulation tend to be tryptophan metabolites such as kynurenine and kynurenic acid. Various studies have demonstrated that the immunosuppressive effects of ASCs can be changed by specific inhibition of these soluble factors. Furthermore, the expression of cytokines such as IL-6 can downregulate expression of MHC-II and CD86 on dendritic cells (DCs) and prohibit their differentiation, further suppressing the immune response by hampering antigen presentation and/or costimulatory signaling of APCs. However, it is still not clear whether a direct cell-to-cell contact is required for this suppression [199]. While some studies confirmed the pivotal role of such contacts, some others revealed that ASCs exert their immunosuppressive properties without a cell–cell contact [194,199,200]. The immunosuppressive properties of ASCs with their immune-privileged status make these cells suitable for allogeneic and xenogeneic transplants usage without the need for immunosuppressants [190,201,202]. The potency of their immunosuppressive ability is demonstrated by the fact that ASCs have been trialed as a treatment for graft-versus-host disease and transplant rejection [203,204,205]. The advantage of using allogeneic ASCs is that cells from a single donor can potentially be employed to treat all the patients in a trial, removing donor variance. The cells must be expanded in culture to provide the numbers required for the use ASCs from a single donor in multiple patients in clinical trial. However, this process can have a significant result on ASCs bioactivity [206]. The US Food and Drug Administration (FDA) has proposed standard assays that are required to assess the bioactivity of the cells utilized in clinical trials. In that way, the therapeutic benefit can be connected to the potency of the cells used, and also allow for comparisons across clinical trials that have employed cells from different donors at different stages of expansion.

BMSCs are another immunoregulatory MSCs. The immunomodulatory properties of BMSCs are facilitated by their interactions with immune cells such as macrophages, T cells, B cells, and DCs in a context and microenvironment-dependent manner. These cells can also inhibit natural killer (NK) cells activity, B cell proliferation, and DC function and differentiation. Moreover, BMSCs are considered to immunosuppress the local environment by secretions of cytokines and growth factors and cell–cell interaction [207]. For instance, soluble factors such as growth factors and cytokines, namely PGE2, IDO, IL-6, and M-CSF, have been evaluated in various clinical studies and the cell-based properties have been explored in many T cell-mediated diseases. Moreover, the evidence demonstrated that both undifferentiated and differentiated BMSCs have a suppressive impression on mitogen-stimulated and alloantigen lymphocyte proliferation followed by a concomitant reduction in the production of pro-inflammatory cytokines such as TNF-α and IFN-γ [208,209]. Therefore, the clinical applications of human BMSCs are ranging from transplantation, immune-related disorders including autoimmune disorders and cell replacement [210].

Functional characterization of BMSCs and ASCs has revealed that both cell types are able to suppress lymphocyte reactivity in mixed lymphocyte response (MLR) assays and decrease the production of inflammatory cytokines in vivo [200,202]. Particularly, additional recent studies reported that delivering ASCs in polyhydroxybutyrate-co-hydroxy valerate constructs achieves a similar outcome to BMSCs role in wound healing and may be more clinically appropriate since the yield following isolation is much higher than BMSCs [177,211].

## 7. Immunomodulatory Skin Scaffolds

Recently, a tremendous effort has been made to design biomaterials with appropriate mechanical, chemical, and biological properties, which closely interact with the host tissue. However, engineering such biomaterials requires an in-depth understanding of how the host inflammatory responses are regulated during the wound healing of implanted biomaterials [212]. Hence, numerous studies have focused on the development of immunomodulatory biomaterials that reduce the inflammation phase of skin healing and thus, diminish scar formation [68,213,214]. The main targets of the immunomodulatory biomaterials are immune cells such as leukocytes, in particular neutrophils, macrophages, mast cells, and T cells [68].

Different physical and chemical properties of the biomaterial such as stiffness, topography, roughness, pore size and pore distribution, degradation rate and its debris, surface charge, ligand presentation, and surface functional groups influence the behavior of host cells [215,216]. However, the effects of such biophysical and biochemical characteristics on immune cells, especially when a skin substitute is implanted, are still not elucidated. A biomaterial should be designed to minimize the deleterious host body responses [217,218,219,220,221]. The host immune system response after implantation of an engineered skin substitute is called foreign body reaction (FBR), which can cause significant problems for patients through excessive inflammation and adverse effects on tissue repair processes. Therefore, controlling the biomaterial interaction with the host tissue or FBR is of crucial importance in the field of regenerative medicine and tissue engineering [222,223,224]. In this respect, the term “bioinert implant” refers to any material that is placed in the human body and demonstrates a minimal interaction with its surrounding tissue. Specifically, an acellular fibrous capsule is formed at the interface between tissues and bioinert biomaterials [225]. However, novel biomaterials are being designed to stimulate specific cellular responses at the cellular level to trigger desired immunological outcomes, thereby supporting the wound healing process [212,226].

Consideration of FBR is important as it can impact the biocompatibility of the implanted biomaterial and can expressively impact short- and long-term tissue reactions with tissue-engineered substitutes containing cells, proteins, and other biological components for use in tissue engineering and regenerative medicine [227]. The FBR can be characterized by the presence of different immune cells, especially macrophages at the tissue-material interface [228]. Additionally, as the macrophages are already presented at the wound site and have a prominent role in the wound healing process, such as release of enzymes important for tissue restructuring and of cytokines and growth factors inducing migration and proliferation of fibroblasts, the effect of the microenvironment produced by scaffolds on these cells should be completely evaluated [228,229].

When a biomaterial is implanted into a vascularized wound bed, the natural innate body response is that plasma proteins are immediately adsorbed onto the implanted biomaterial surface. Factor XII (FXII) and tissue factor (TF) are the initiators of the intrinsic and extrinsic system of the coagulation cascade, respectively, leading to the formation of a blood clot. This leads to infiltration and adherence of cells such as platelets, monocytes, and macrophages through the interaction of adhesion receptors with the adsorbed proteins [212,227]. Adhered cells release growth factors and chemokines, which are able to recruit cells of the innate immune system to the injury/implantation site. Finally, deposition and organization of collagen matrix arise from fibroblasts and MSC activities [230].

**Figure 7:**
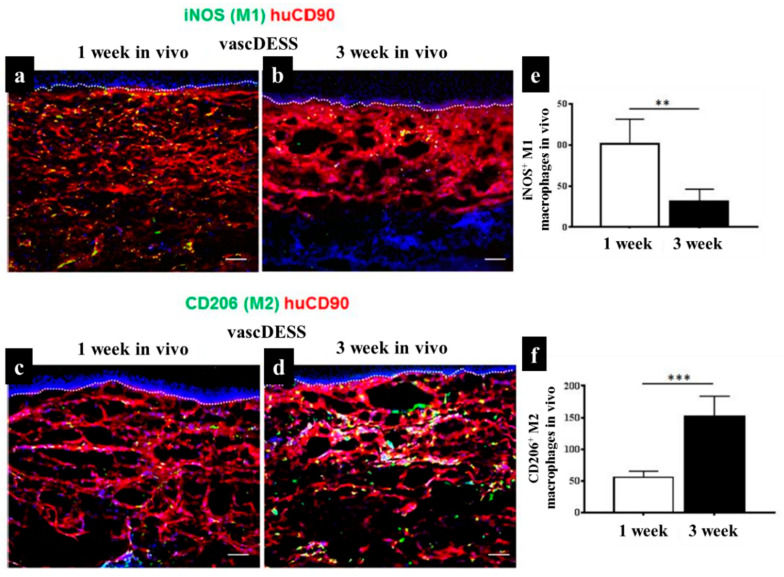
Infiltration of distinct subsets of macrophages into vascDESS in vivo. (**a**,**b**) Infiltration of iNOS M1 macrophages into vascDESS in vivo. Double-label immunofluorescence for iNOS macrophages (green) and human CD90 (red) in vascDESS at 1 and 3 weeks of post-transplantation. (**c**,**d**) Expression of CD206 in transplanted vascDESS in vivo. M2 macrophages were detected with an antibody against CD206 (green) within the human CD90-stained neodermis (red). CD206 macrophage (M2) density was increased in vascDESS after 3 weeks as compared to 1 week in vivo. (**e**,**f**) The quantification of iNOS and CD206 density in transplanted skin analogs at 1 and 3 weeks in vivo (** *p* < 0.01,*** *p* < 0.001) [158].

As mentioned in previous sections, when macrophages migrate into inflamed tissues, they become activated and exhibit a spectrum of polarization states associated with their functional diversity, eventually activated into the pro-inflammatory M1 and the anti-inflammatory M2 states [231]. In the case of skin tissue substitute implantation, although the initial presence of M1-macrophages supports to start a necessary inflammatory reaction, a prolonged M1-macrophage presence causes a severe FBR and fibrous encapsulation leading to chronic inflammation and failure of biomaterial interaction and integration [216]. Therefore, there should be a short M1-macrophage stage for regenerative approaches, when the target is to reproduce lost tissue and avoid scar tissue formation. M2-macrophages are activated by granulocytes such as mast cells and basophils. M2-macrophages continuously release anti-inflammatory cytokines, display a high level of iron export assisting tissue remodeling [60,232,233]. The existence of these anti-inflammatory cytokines and the tissue remodeling reaction can improve the vascularization of regenerative tissue substitutes by preventing fibrous tissue formation, but promoting the integration of the biomaterials [234]. Macrophages constantly sense signals from their environment through various biochemical and biophysical cues and might change their polarization status accordingly [234]. In an animal study, we engineered a vascularized human dermo-epidermal skin substitute (vascDESS) in vitro and transplanted it on the back of immuno-incompetent rats to evaluate M1 and M2 polarization of macrophages during the wound healing process [158]. Rat M1 macrophages were quantified in vascDESS after 1 and 3 weeks of post-transplantation using a specific antibody for iNOS (green) and engineered human dermal compartment of skin analogs was stained with human CD90 antibody (red) (Figure 7). M1 macrophages were present at high levels and scattered throughout the whole dermal part of skin analogs at 1 week whereas only some cells were detected at 3 weeks. Moreover, vascDESS demonstrated a moderate number of rat macrophages of M2 phenotype at 1 week. In contrast, the transplants were heavily infiltrated by those macrophages at 3 weeks (Figure 7).

Macrophages exhibit a heterogeneous and temporally regulated polarization during skin wound healing and the phenotype changes during healing from a more pro-inflammatory (M1) profile in early stages after injury, to a less inflammatory, pro-healing (M2) phenotype in later phases. Many studies have proved that a high M2:M1 ratio in the surrounding environment of implanted biomaterials results in better remodeling outcomes [235,236,237,238]. During skin wound healing M1 macrophages are replaced by M2 polarized cells [239]. Both M1 and M2 cells exert specific functions in this process [9,240]. It has been suggested that the long-term presence of M1 macrophages leads prolonged inflammation phase and impairs finally the wound healing outcome [8,158,232,239,241]. The same is true for M2 cells, which can lead over time to the formation of detrimental foreign body giant cells [227,242]. Therefore, the control of the M1:M2: ratio is vitally important when designing an immune-active scaffold to promote tissue remodeling as well as its integration and regeneration in vivo [158].

Therefore, a considerable effort has been made to employ particular ECM components and natural polymers, which are able to mimic the ECM structure to design appropriate immunomodulatory biomaterials for tissue engineering and regenerative medicine. By modulating the immune system, these biomaterials could be mainly applied to treat a broad spectrum of immune-related skin diseases in the future. Table 2 presents the summary of some papers conducted on natural hydrogels for skin immunomodulation.

### 7.1. Immunomodulation of Naturally Derived ECM Skin Scaffolds

The ECM provides a structural basis for cells and has a significant role in regulating cell functions. ECM is composed of proteins, glycoproteins, and polysaccharides. Recently, it has been proved that the ECM contains bioactive motifs which are able to modulate immune responses directly as they are recognized by specific cell surface receptors [265]. Moreover, cells can regulate their ECM microenvironment by synthesizing a new matrix component and digesting an existing matrix to alter its modulatory effects [266]. The degradation of the ECM components such as collagens can modulate the behavior of cells [267]. With regard to this, the ECM components have natural immunomodulatory domains that can bind specifically the surface receptors of immune cells, allowing their adhesion and activation. Thus, specific ECM proteins, glycans, and peptides can be used in scaffolds to mimic the natural regulatory role of distinct matrix components on the immune system to improve the longevity and functionality of implants [226,268,269].

Hence, the immunomodulatory effects of various ECM bioscaffolds, which are derived by decellularization of native tissues, have been broadly studied [226,267,268,269,270,271,272]. In this respect, the grade of decellularization of the donor tissue is a critical factor, which can affect the immunomodulation property of ECM, in particular, to elicit an anti-inflammatory macrophage/T cell (M2-like/Th2-like) host response. Additionally, other parameters including cell removal techniques, age of tissue, chemical crosslinking degree and agent, and terminal sterilization method can influence their immunomodulatory properties and markedly affect the host response to ECM bioscaffolds [226,270]. In a particular study, Huleihel et al. [271] demonstrated that macrophages could react differently to ECM scaffolds depending on the source of ECM and processing procedures. Accordingly, in another study, it has been shown that chemical crosslinking of the porcine ECM with water-soluble carbodiimide caused a switch from an M2 dominant to an M1 dominant profile [272]. Remarkably, the autologous tissue graft displayed an M2 response early followed by a duality of the M1 and M2 reaction, which can be considered as a result of pro-inflammatory cytokines produced by dead cells or damage associated molecules released by dying cells within the tissue substitute. In this regard, the M2 polarization profile was linked to remodeling and maturation, while the M1 phenotype profile was associated with chronic inflammation [268].

In general, to synthesize the ECM-based scaffold for immunomodulation in regenerative medicine, native tissues or full-length ECM molecules are utilized as building units for bioscaffolds. Tissue substitutes composed of ECM are typically derived from xenogeneic tissues and have demonstrated considerable success in developing constructive and functional tissue remodeling in multiple anatomic sites in both preclinical and clinical studies. ECM scaffolds are synthesized by techniques that remove essentially all cellular fragments such as xenogeneic antigens that would normally elicit a pro-inflammatory response, lyophilization, and further digestion. It is served as an inductive niche to impact cell behavior and the downstream tissue remodeling response [226]. Clinically, these decellularized matrices have been used to support the healing of bone, muscle, tendon, breast, heart, and skin [273]. These materials compositions are highly dependent on the origin tissue and processing method. The matrices’ composition and thereby their physical and biochemical properties influence their immunomodulatory properties [267]. Generally, the mechanisms by which ECM scaffolds can promote tissue regeneration and remodeling include mechanical support, controllable degradation rate and release of bioactive molecules, recruitment and differentiation of endogenous stem/progenitor cells, and modulation of the immune response toward an anti-inflammatory phenotype [270]. However, the immunomodulatory properties of decellularized matrices can be varied based on antigen removal technique, microstructure, age, and tissue source. Thus, it is difficult to control the precise molecular composition of these scaffolds, as well as contaminants in materials derived from natural sources. Finally, an engineered hydrogel with engineered characteristics can provide better control over the matrix composition and properties.

### 7.2. Immunomodulatory Natural Hydrogels for Skin Wounds

Hydrogels are highly hydrated three-dimensional (3D) structures consisting of physically (e.g., ionic) or chemically (e.g., photopolymerization) crosslinked bonds of hydrophilic polymers [17,274,275,276,277]. The hydrophilic nature and high swelling ratio make hydrogels permeable to oxygen, metabolites, nutrients, and cellular waste. Hydrogels have become vastly popular in regenerative medicine especially in skin engineering, due to their biocompatibility, flexibility, surface property, and a broad spectrum of choice of base material. However, the advantage of hydrogels such as biocompatibility, cell adhesion, enzymatic and hydrolytic degradability, minimal inflammatory response, and ability to stimulate a specific cellular response can heavily depend on polymer choice and its chemical and physical characteristics [7,275,277].

There are various natural hydrogels that are able to mimic ECM structure and have been utilized to control the immune system and conduct skin regeneration [278,279,280].

Moreover, biochemical and biophysical signals from injected/implanted hydrogels can affect immune cell behavior and consequently change the M2:M1 ratio [281]. Thereafter, in the field of regenerative medicine, the selection of an appropriate “immuno-informed” hydrogel to enrich positive tissue remodeling is vitally important. Over the last decade, many studies have been conducted to examine the immunomodulatory properties of hydrogels and revealed that hydrogels could modify inflammatory pathways. It is known that many different factors including the crosslinking degree, degradation rate, hydrophilicity degree, surface chemistry, and energy, size and shape of the hydrogel are crucial factors that can influence immunostimulatory signals [214].

For instance, hydrophilic polymers and neutrally charged hydrogels have been described to stimulate less macrophage and foreign body giant cell attachment in comparison with hydrophobic and positively/negatively surface charged biomaterials. Furthermore, macrophages and foreign body giant cells attaching to the hydrophilic surfaces secrete fewer cytokines than those attached to hydrophobic surfaces [282,283]. Further, the surface topography of hydrogels is one of the crucial factors to control immune responses [284]. However, the effect of surface topography and morphology of hydrogels on the immunomodulatory responses such as macrophage polarization is not well described. Interestingly, in the study by Singh et al. [235] a gelatin methacryloyl (GelMA) hydrogel platform was used to determine whether micropatterned surfaces can modulate the phenotype and function of macrophages by cytokine profile, surface marker expression, morphology, phagocytosis, and gene microarrays evaluations. The findings indicated that micropatterns induce distinctive gene expression profiles in human macrophages cultured on microgrooves and micropillars. It was observed that significant changes occurred in genes associated with primary metabolic procedures such as protein trafficking, DNA repair, transcription, translation, and cell survival. However, using conventional phenotyping methods, based on surface marker expression and cytokine profile, could not distinguish between the different physicochemical conditions, and demonstrated no significant shift in cell activation toward M1 or M2 phenotypes.

On the other hand, hydrogel composition plays a critical role in cell behavior and can alter the M2:M1 ratio. Hence, various types of hydrogels possess specific characteristics, which are linked to danger-associated molecular patterns or safe molecular patterns for immune body system. For instance, the immune system becomes usually activated by the cyclic patterns of hydrogel chains and hydrophobic sections of components [285,286]. In a comprehensive study, human-derived DCs were seeded on natural hydrogels including agarose, chitosan, hyaluronic acid, and alginate to assess DCs maturation upon in vitro culture [287]. A growth in the expression of several cell surface markers including costimulatory molecules such as CD40, CD80, and CD86, and MHC class II molecules such as HLA-DR and HLA-DQ, and a marker of mature DCs, CD83, can represent the maturation of DCs [288,289]. DCs cultured on chitosan hydrogel increased expression levels of CD86, CD40, and HLA-DQ, here as DCs on alginate or hyaluronic acid hydrogel films decreased their expression levels of these same molecules.

Thus, it can be concluded that the influence of hydrogels on DC maturation, and the associated adjuvant effect, is a novel biocompatibility selection and design criteria for immunomodulatory hydrogels.

#### 7.2.1. Collagen

In the naturally derived ECMs, collagen is the most abundant ECM component in the body. There are 29 different types of collagen and all of them form a distinctive right-handed triple helix structure. They also comprise a large family of proteins with characteristic functions in the ECM structure [290,291]. Collagens types I–III, V, and XI have fibrillar quaternary architectures which suit them for biomaterials applications. Immune cells express various receptors such as integrins, discoidin domain receptors DDR1 and 2, and leukocyte-associated immunoglobulin-like receptor-1 (LAIR-1) of the leukocyte receptor compound that can bind to collagen directly [266]. Collagens I–III and XVII are high-affinity ligands for LAIR-1 receptors on immune cells, where binding prevents degranulation of peripheral basophils, and more generally suppressed immune cell activity [246,291]. In a study by Masry et al. [247], a stabilized, acellular ECM equine pericardial collagen matrix (sPCM) was used as wound dressing to examine its effect on macrophage function and epithelialization. They revealed sPCM was efficient for resolving post-wound inflammation quickly, as indicated by elevated levels of IL-0, arginase-, and VEGF, and lowering of IL-β and TNF-. The sPCM stimulated apoptotic cell uptake (efferocytosis) in murine wound macrophages three days after wound dressing application, a main functional role of macrophages in early wound healing [266]. Moreover, sPCM could accelerate wound re-epithelialization and wound closure by increased collagen deposition in comparison with the control group which was a polycarbonate mesh.

Different commercially available decellularized collagen-based matrices were applied as wound dressings and have been subsequently also studied in terms of the elicited immune response properties. For example, Integra is one of many off-the-shelf acellular matrices for the regeneration of dermal tissue and is composed of a porous scaffold of crosslinked bovine tendon collagen type I and III and glycosaminoglycan [7,17]. Integra is an acellular bi-layer substitute composed of acellular collagen with a removable semipermeable silicone layer acting as a temporary epidermis maintaining moisture and preventing infection. It was found that Integra can decrease tissue inflammation, while increasing the scavenger M2-specific receptor CD163 and decreasing TNFα protein expression in both primary human and THP-1 macrophages when compared with AlloMend (human dermis-derived collagen and elastin scaffold), Pri-Matrix (fetal bovine dermis-derived scaffold, containing type I and III collagens), and Oasis (porcine small intestine submucosa derived scaffold, containing elastin, glycosaminoglycans, proteoglycans, glycoproteins, and collagens I–III) [292].

Additionally, in a study by Angelis et al. [293] the skin regeneration and immunomodulatory characteristics of two different acellular double-layer dermal substitutes, namely Nevelia (native collagen type I and a silicone sheet) and Integra, were compared in patients (n = 30) with post-traumatic skin defects. Their results revealed that both dermal substitutes demonstrated a positive effect on the quality and functionality of skin reconstruction. However, the Nevelia scaffold showed more rapid skin regeneration in terms of epidermal proliferation, dermal renewal, and vascularization.

**Figure 8:**
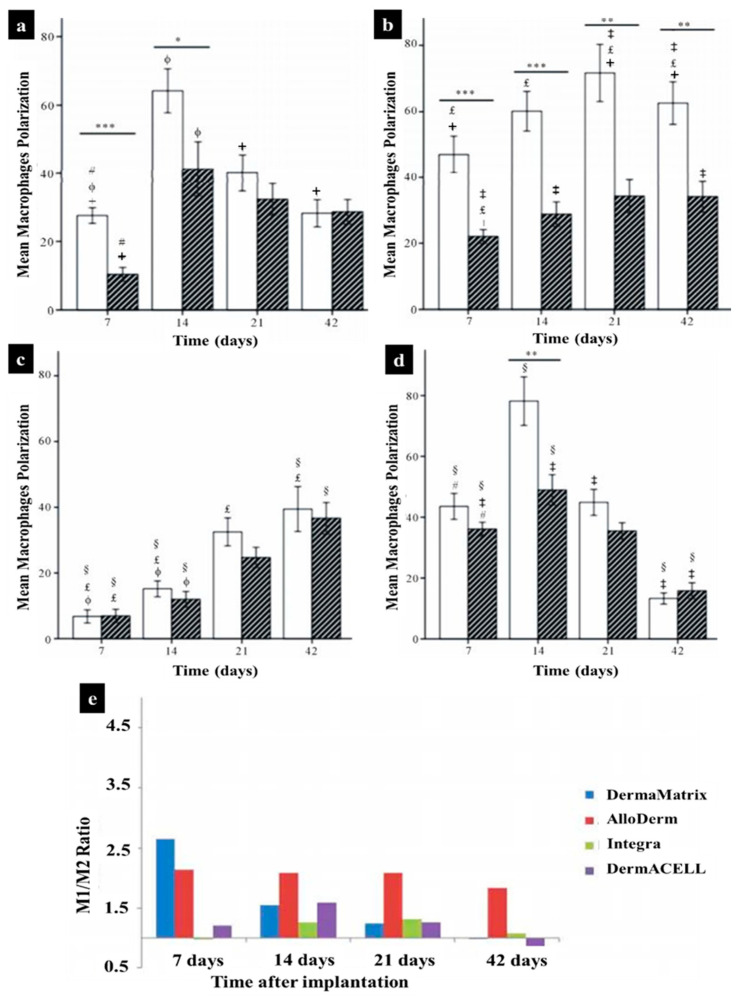
Statistics showing the mean macrophage polarization after implantation of DermaMatrix (**a**), AlloDerm (**b**), Integra (**c**), DermACELL, and (**d**) in adult rats. M1 phenotype represented by white bars and M2 with hatched bars. Comparison between M1 and M2 phenotype at each timepoint; * *p* < 0.05, ** *p* < 0.01, *** *p* < 0.001. (**e**) The ratios of M1:M2 macrophages for dermal matrices at each timepoint (+ DermaMatrix^®^ vs. AlloDerm^®^, ɸ DermaMatrix^®^ vs. Integra^®^, # DermaMatrix^®^ vs. DermACELL^®^, £ AlloDerm^®^ vs. Integra^®^, ‡ AlloDerm^®^ vs. DermACELL^®^, § Integra^®^ vs. DermACELL^®^) [294].

Distinct ECM-like materials in skin substitutes can promote a switch from a predominant M1 macrophage cell population to a population enriched for macrophage M2 cells after 7 to 14 days following implantation [230,231,242]. Recently, in an animal study by Agrawal et al. [294] macrophage phenotype and tissue remodeling elicited by DermaMatrix, AlloDerm, Integra, and DermACELL dermal matrices were investigated. Based on immunohistochemistry findings, all skin matrices presented a normal and similar curve shape for distribution of macrophages stained by CD68. A quantitative analysis of the macrophage phenotypes in AlloDerm showed they were mainly M1 at all timepoints. On the other hand, Integra exhibited a mixed M1:M2 population of macrophages at all days with no considerable differences. However, Integra demonstrated an increased M2:M1 ratio on days 7 and 42 and an increased M1:M2 ratio on days 14 to 21. Further, macrophages in DermaMatrix were predominantly M1 at 7 and 14 days post-implantation, with a mixed M1/M2 population at 21 and 42 days. Interestingly, the M1:M2 ratio for this scaffold progressively changed from the M1 toward the M2 phenotype over time. For the DermACELL scaffold, although the M1:M2 ratio experience an increase from day 7 to day 21, it declined gradually to an M2 predominant response at day 42, as illustrated in Figure 8.

#### 7.2.2. Fibrin

Fibrin plays an important role in the natural wound healing process and it is the main component of the hemostatic clot [249]. Platelets adhere to fibrin through αiibβ3 (GP2b3a), ɑvβ1, and P-selectin and thereby stabilizing the clot [295]. Therefore, fibrin-based hydrogels are one of the most appreciated materials in wound healing approaches and can be used as a promising immunomodulatory component for wound healing. In early studies, fibrin matrix was produced in vitro through the mixing of purified fibrinogen with activated thrombin to evaluate the effects of fibrin on immune cell function [248,250,296]. The findings indicated that macrophage motility was inhibited by increasing the fibrinogen or factor-XIII crosslinker concentration in fibrin matrices, whereas fibroblasts could migrate more effectively through factor-XIII crosslinked fibrin gels.

In a more recent study, Hsieh et al. [251] cultured the primary murine bone marrow-derived macrophages (BMDMs) on fibrin hydrogels. The group revealed that the soluble inflammatory TNF-α cytokine secretion by the macrophages in response to LPS/IFN-γ was significantly decreased as compared to the control group in which cells were cultured on tissue culture plastic. In addition, this study demonstrated the application of fibrin-based hydrogels on porcine burn wounds resulted in macrophage and neutrophil recruitment reduction, demonstrating extended pro-healing effects of fibrin in vivo injury models [250]. Moreover, fibrin and the hemostasis cascade impacted the adaptive immune activity via activation of DCs trafficking and sequestration of lymphangiogenic growth factors [252]. In addition, fibrin can bind vascular endothelial growth factor (VEGF)-C during the wound healing process, and this sequestration is necessary for lymphangiogenesis or the formation of new lymphatic vessels, which plays a key role in maintaining communication between antigen-presenting cells and the adaptive immune compartment [297].

Altogether, fibrin hydrogels provide a temporary matrix and significantly elicit inflammatory and anti-inflammatory responses via cytokine secretions, increasing the initial inflammatory reactions and accelerating the transition to the later proliferative and remodeling phases of the wound healing process [298,299]. Additionally, as described above, matrix features such as mechanical properties and architecture can also affect immune cells function. However, the exact elements affecting the way of fibrin immunoregulation are not fully investigated and more investigations are desirable to reveal and characterize the interactions between the provisional matrix and the immune cells.

#### 7.2.3. Hyaluronic Acid

The glycosaminoglycan hyaluronic acid (HA) is a nonbranched polyanionic polysaccharide with various molecular weights (MW) ranging from 1 kDa to 2 MDa. HA is primarily limited to the skin and musculoskeletal tissue and it associates with proteoglycan aggrecan to form aggregates [253]. HA is not sulfated and undergoes a rapid turnover in the body, mainly during wound healing [253]. It is also crucial for many cellular and tissue functions. HA is in clinical use for decades, in various applications such as cosmetics, drug delivery, and wound healing [254,300]. Structural and biochemical properties of HA affect cell signaling during different stages of development or tissue repair and immune cell responses, depending on its MW. The high-MW HA is immunosuppressive while the low-MW HA enhances an inflammatory response [267,301]. Additionally, according to the significant effects of high-MW HA in wound healing, it has been employed as immunomodulation hydrogel for the treatment of chronic wounds [302].

Overall, macrophages undergo phenotypic changes dependent on the molecular weight of hyaluronan that corresponds to either pro-inflammatory response for low-MW HA or pro-resolving response for high-MW HA. High-MW HA delays inflammation and decreases the regeneration of inflammatory cytokines by various cell types. This trend is associated with the interaction between high-MW HA and CD44, which is the main cell surface HA-binding transmembrane glycoprotein and it is able to transduce signals from the ECM, which influence cell activation, growth, and differentiation, and contributes to immune homeostasis through the preservation of Th1 memory cells [303]. By contrast, low-MW HA mediates the activation and maturation of DCs, stimulates the release of pro-inflammatory cytokines including IL-1ß, TNF-a, IL-6, and IL-12 by multiple cell types, and cell trafficking and induces endothelial cell proliferation. Therefore, low-MW HA can act as a pro-inflammatory molecular pattern [254].

#### 7.2.4. Chitosan

Chitosan hydrogel, a natural polysaccharide, serves a range of distinct properties including biocompatibility, engineerable degradation rate and mechanical properties, bioactivity, nonantigenicity, adhesiveness, and antibacterial and hemostatic characteristics, which suits this biomaterial for wound healing applications [304,305]. Moreover, multifunctional and immunomodulatory features of chitosan have attracted much attention due to its ability to induce innate immune cells to release a wide range of growth factors, pro- and anti-inflammatory cytokines, chemokines, and bioactive lipids [255]. Chitosan hydrogel can employ the inflammatory cells including leukocytes and macrophages to the wound site and thereby promotes granulation of injured tissue and promotes wound healing [256]. The mechanism of macrophage stimulation in contact with chitosan hydrogels includes mannose receptor-mediated phagocytosis. The mannose receptor is favorably controlled on macrophages, improving the interaction with appropriate ligands including chitosan. In addition to the effective role on the inflammation step, chitosan can accelerate wound contraction by some modifications in its backbone chain or by incorporation of biomolecules [257]. In this regard, Moura et al. [258] employed a chitosan-based dressing to heal early diabetic wounds in mice after functionalization with 5-methyl pyrrolidinone. Their finding demonstrated the wound healing process was accelerated due to a decrease in the inflammatory cells, and TNF-α and MMP-9 levels. Moreover, Ashouri et al. [237] combined the chitosan with aloe vera to investigate the role of prepared aloe vera/chitosan nanohydrogels on macrophage polarization in wound healing and the balance between M1 and M2 macrophages. Aloe vera/chitosan nanohydrogels could decrease M1 after 3 days and increase M2 after 14 days, which eventuated in accelerated and optimal wound repair.

## 8. Conclusions and Future Direction

Recent findings have greatly improved our understanding of the roles of the immune system in acute wound healing. Accordingly, the immune response is either dysregulated in chronic wounds or leads to undesirable scar development during skin wound healing. Notably, macrophages are key players in those processes, in particular through their polarization into different phenotypes during wound healing.

Further studies on activation pathways could improve our understanding of how to influence the macrophage polarization to finally improve wound healing outcomes. Additionally, regulatory T cells have been also shown to have proregenerative properties in some tissues.

Ultimately, translating the current knowledge of immunomodulatory stem cell properties hinges on the development of appropriate biomaterials. Currently, there are different immunomodulatory scaffolds showing specific properties that can be applied for skin tissue engineering. However, in addition to their in-depth physical and chemical characterization, also their impact on the immune system response should be considered. In this respect, the incorporation of BMSCs and ASCs in skin substitutes can alter immune responses and affect macrophage phenotype. Ideally, the immune response in the implanted skin substitute should be stimulated toward an M2 phenotype for improved matrix remodeling and tissue regeneration.

Furthermore, the skin substitutes should be tissue-engineered in a way that they do not trigger an exaggerated foreign body response. Moreover, the degradation products and remodeling of the biomaterials need to be carefully controlled as they represent important pro-/anti-inflammatory cues.

Finally, new insights of how MSCs immunomodulate biomaterial properties will permit the design of appropriate scaffolds for skin substitutes and predict immune cell response to them.

## Figures and Tables

**Table 1:** Summary of studies implying induced pluripotent stem cells in cutaneous wound healing in a murine model, including cell type, delivery method, animal model, and major findings [96].

Author	Cell Type	Delivery Method	Animal Model	Major Findings
Clayton et al. [111]	hiPSC-derived endothelial cells	Intradermal injection Suspended in medium and Matrigel	Nude mice Nondiabetic	Increased vascularization Accelerated wound closure High collagen deposition, macrophage number, blood vessel density Increased wound perfusion Increased host expression of PECAM, Tie-1, and VEGF
Kim et al. [110]	hiPSC-derived smooth muscle cells and endothelial	Intradermal injection in PBS	Nude mice Nondiabetic	Increased angiogenesis Accelerated wound closure Increased smooth muscle cell migration Increased in vitro VEGF, EGF, and FGF-4
Shen et al. [116]	hiPSC early vascular cells	Topical application Acrylated hyaluronic acid hydrogels	Nude mice STZ diabetic	Accelerated wound closure Increased wound perfusion No significant difference between healthy and diabetic donor derived cells Increased blood vessel density
Tan et al. [117]	hiPSC-derived endothelial cells	Topical application Electrospun PCL/gelatin scaffolds	FVB/N mice Nondiabetic	Increased angiogenesis compared Increased cell survival in scaffolds compared to cellular injections Increased arteriole density in scaffold group
Kashpur et al. [118]	hiPSC-derived fibroblasts	Topical application Polyethylene terephthalate membrane self-assembled tissues	Nude mice STZ diabetic	Accelerated wound healing with hiPSC-derived fibroblasts from DFU compared to primary cells No difference in gene expression between hiPSC-derived fibroblasts derived from healthy and diabetic patients
Nakayama et al. [119]	hiPSC-MSC	Intravenous injection	Nude mice Nondiabetic	Accelerated wound healing as measured by epithelialization after IV delivery of 1 × 10^6^ cells
Kobayashi et al. [120]	hiPSC-derived extracellular vesicles	Intradermal injection + topical application in PBS	C57 mice db/db diabetic	Increased angiogenesis Accelerated wound healing Increased fibroblasts migration and replication, in vitro

**Table 2:** Summary of research studies conducted on natural hydrogel-based materials used for immunomodulation of skin regeneration.

Hydrogel Type	Main Characteristic	Immunomodulatory Role in Skin Regeneration	Ref.
Decellularized ECM	Containing proteins, glycoproteins, and polysaccharides	Binding to the specific surface receptors of immune cells, the ECM composition can affect immunomodulation, promoting anti-inflammatory phenotype polarization in some cases	[230,243,244,245]
Collagen	Protein-based material and the most abundant ECM component	Binding to LAIR-1 receptors on immune cells, suppressing immune cell activity, and resolving post-wound inflammation	[246,247]
Fibrin	The main component of the haemostatic clot	Decreasing TNF-α cytokine secretion, decreasing macrophage motility, reduction of neutrophil recruitment, and extending pro-healing effects	[248,249,250,251,252]
Hyaluronic acid	Glycosaminoglycans material with various MWs	Modulation of leukocyte function, immunomodulatory effect dependent on MW, suppressing immune cell activity by high-MW HA, enhancing an inflammatory by low-MW HA, delaying inflammation by High-MW HA	[253,254]
Chitosan	A natural polysaccharide	Employing leukocytes and macrophages to the wound site, decreasing the inflammatory cells, and TNF-α and MMP-9 levels, and affecting macrophage polarization	[255,256,257,258]
Carrageenan	A natural marine polysaccharide from red seaweed	Stimulating IL-10 expression, prohibiting cytotoxic T cell responses, and delaying neutrophil activation	[259,260,261]
L-arginine	Amino acid-based material	Decreasing nitric oxide production, stimulating macrophages to express both TNF-α and NO in combination with chitosan	[262,263,264]

## Data Availability

The study did not report any data.

## References

[1] Vig K., Chaudhari A., Tripathi S., Dixit S., Sahu R., Pillai S., Dennis V.A., Singh S.R. (2017). Advances in Skin Regeneration Using Tissue Engineering. Int. J. Mol. Sci..

[2] Gaur M., Dobke M., Lunyak V.V., Piatelli A., Zavan B. (2017). Molecular Sciences Mesenchymal Stem Cells from Adipose Tissue in Clinical Applications for Dermatological Indications and Skin Aging. Int. J. Mol. Sci..

[3] Roupé K., Nybo M., Sjöbring U., Alberius P. (2010). Injury is a major inducer of epidermal innate immune responses during wound healing. J. Investig. Dermatol..

[4] Klar A.S., Güven S., Biedermann T., Luginbühl J., Böttcher-Haberzeth S., Meuli-Simmen C., Meuli M., Martin I., Scherberich A., Reichmann E. (2014). Tissue-engineered dermo-epidermal skin grafts prevascularized with adipose-derived cells. Biomaterials.

[5] Böttcher-Haberzeth S., Klar A.S., Biedermann T., Schiestl C., Meuli-Simmen C., Reichmann E., Meuli M. (2013). “Trooping the color”: Restoring the original donor skin color by addition of melanocytes to bioengineered skin analogs. Pediatr. Surg. Int..

[6] Klar A.S.S., Böttcher-Haberzeth S., Biedermann T., Clemens S., Ernst R., Meuli M. (2014). Analysis of blood and lymph vascularization patterns in tissue-engineered human dermo-epidermal skin analogs of different pigmentation. Pediatr. Surg. Int..

[7] Tavakoli S., Klar A.S. (2021). Bioengineered Skin Substitutes: Advances and Future Trends. Appl. Sci..

[8] Klar A.S., Güven S., Zimoch J., Zapiórkowska N.A., Biedermann T., Böttcher-Haberzeth S., Meuli-Simmen C., Martin I., Scherberich A., Reichmann E. (2016). Characterization of vasculogenic potential of human adipose-derived endothelial cells in a three-dimensional vascularized skin substitute. Pediatr. Surg. Int..

[9] Klar A.S., Böttcher-Haberzeth S., Biedermann T., Michalak K., Kisiel M., Reichmann E., Meuli M. (2014). Differential expression of granulocyte, macrophage, and hypoxia markers during early and late wound healing stages following transplantation of tissue-engineered skin substitutes of human origin. Pediatr. Surg. Int..

[10] Zimoch J., Padial J., Klar A. (2018). Polyisocyanopeptide hydrogels: A novel thermo-responsive hydrogel supporting pre-vascularization and the development of organotypic structures. Acta Biomater..

[11] Halim A., Khoo T., Yussof S. (2010). Biologic and synthetic skin substitutes: An overview. Indian J. Plast Surg..

[12] Klar A.S., Zimoch J., Biedermann T. (2017). The Use of Adipose Derived Cells for Skin Nerve Regeneration-Short Review of Experimental Research. J. Tissue Sci. Eng..

[13] Shevchenko R.V., James S.L.E., James S.L.E. (2010). A review of tissue-engineered skin bioconstructs available for skin reconstruction. J. R. Soc. Interface.

[14] Vacanti J., Lancet R.L. (1999). Tissue engineering: The design and fabrication of living replacement devices for surgical reconstruction and transplantation. Lancet.

[15] Chen M., Przyborowski M. (2009). Stem cells for skin tissue engineering and wound healing. Crit. Rev. Biomed. Eng..

[16] Klar A., Zimoch J., Biedermann T. (2017). Skin tissue engineering: Application of adipose-derived stem cells. Biomed Res. Int..

[17] Tavakoli S., Klar A.S. (2020). Advanced Hydrogels as Wound Dressings. Biomolecules.

[18] Metcalfe A.D., Ferguson M. (2007). Bioengineering skin using mechanisms of regeneration and repair. Biomaterials.

[19] Metcalfe A.D., Ferguson M.W.J. (2007). Tissue engineering of replacement skin: The crossroads of biomaterials, wound healing, embryonic development, stem cells and regeneration. J. R. Soc. Interface.

[20] Clayton K., Vallejo A.F., Davies J., Sirvent S., Polak M.E. (2017). Langerhans cells-programmed by the epidermis. Front. Immunol..

[21] (2017). MA Nilforoushzadeh Dermal Fibroblast Cells: Biology and Function in Skin Regeneration. J. Ski..

[22] Dhivya S., Padma V. (2015). Wound dressings–a review. Biomedicine.

[23] Moore K., McCallion R., Searle R.J., Stacey M.C., Harding K.G. (2006). Prediction and monitoring the therapeutic response of chronic dermal wounds. Int. Wound J..

[24] Lazarus G.S., Cooper D.M., Knighton D.R., Margolis D.J., Percoraro R.E., Rodeheaver G., Robson M.C. (1994). Definitions and guidelines for assessment of wounds and evaluation of healing. Wound Repair Regen..

[25] Boateng J.S., Matthews K.H., Stevens H.N.E., Eccleston G.M. (2008). Wound healing dressings and drug delivery systems: A review. J. Pharm. Sci..

[26] Percival N.J. (2002). Classification of Wounds and their Management. Surgery.

[27] Singer A.J., Clark R.A.F. (1999). Cutaneous Wound Healing. N. Engl. J. Med..

[28] Larson B.J., Longaker M.T., Lorenz H.P. (2010). Scarless fetal wound healing: A basic science review. Plast. Reconstr. Surg..

[29] Guo S., DiPietro L.A. (2010). Factors Affecting Wound Healing. J. Dent. Res..

[30] Käser S.A., Zengaffinen R., Uhlmann M., Glaser C., Maurer C.A. (2014). Primary wound closure with a Limberg flap vs. secondary wound healing after excision of a pilonidal sinus: A multicentre randomised controlled study. Int. J. Colorectal Dis..

[31] Burns P.S. (2001). Burn wound healing and skin substitutes. Burns.

[32] Martin P. (1997). Wound Healing-Aiming for Perfect Skin Regeneration. Science.

[33] Wallace H., Basehore B., Zito P. Wound Healing Phases; StatPearls Publishing, Treasure Island (FL) 2020. https://europepmc.org/books/n/statpearls/article-34001/.

[34] Kloc M., Ghobrial R.M., Wosik J., Lewicka A., Lewicki S., Kubiak J.Z. (2019). Macrophage functions in wound healing. J. Tissue Eng. Regen. Med..

[35] Gilmore M.A. (1991). Phases of wound healing. Dimens Oncol. Nurs..

[36] Wilgus T.A., Roy S., McDaniel J.C. (2013). Neutrophils and Wound Repair: Positive Actions and Negative Reactions. Adv. Wound Care.

[37] Iacob A.T., Drăgan M., Ionescu O.M., Profire L., Ficai A., Andronescu E., Confederat L.G., Lupașcu D. (2020). An Overview of Biopolymeric Electrospun Nanofibers Based on Polysaccharides for Wound Healing Management. Pharmaceutics.

[38] McCartney-Francis N.L., Wahl S.M. (2001). TGF-β and macrophages in the rise and fall of inflammation. TGF-β and Related Cytokines in Inflammation.

[39] Turner M., Nedjai B., Hurst T. (2014). Cytokines and chemokines: At the crossroads of cell signalling and inflammatory disease. Biochim. Biophys. Acta.

[40] Werner S., Krieg T., Smola H. (2007). Keratinocyte–fibroblast interactions in wound healing. Keratinocyte–Fibroblast Interact. Wound Health.

[41] Nelson E.A. (2003). Nutrition for optimum wound healing. Nurs. Stand..

[42] Gordillo G.M., Sen C.K. (2003). Revisiting the essential role of oxygen in wound healing. Am. J. Surg..

[43] Branton M.H., Kopp J.B. (1999). TGF-β and fibrosis. Microbes. Infect..

[44] Phan S.H. (2008). Biology of Fibroblasts and Myofibroblasts. Proc. Am. Thorac. Soc..

[45] Van De Water L., Varney S., Tomasek J.J. (2013). Mechanoregulation of the Myofibroblast in Wound Contraction, Scarring, and Fibrosis: Opportunities for New Therapeutic Intervention. Adv. Wound Care.

[46] Darby I., Laverdet B. (2014). Fibroblasts and myofibroblasts in wound healing. Clin. Cosmet. Investig. Dermatol..

[47] Basu A., Kligman L.H., Samulewicz S.J., Howe C.C. (2001). Impaired wound healing in mice deficient in a matricellular protein SPARC (osteonectin, BM-40). BMC Cell Biol..

[48] Chen D., Hao H., Fu X. (2016). Insight into reepithelialization: How do mesenchymal stem cells perform?. Stem Cells Int..

[49] Santoro M.M., Gaudino G., Santoro M.M. (2005). Cellular and molecular facets of keratinocyte reepithelization during wound healing. Exp. Cell Res..

[50] Martins V.L., Caley M., O’toole E.A. (2013). Matrix metalloproteinases and epidermal wound repair. Cell Tissue Res..

[51] Nguyen T.T., Mobashery S., Chang M. (2016). Roles of Matrix Metalloproteinases in Cutaneous Wound Healing. Wound Health New Insights Into Anc. Chall..

[52] Michopoulou A., Bernard C., Lyon U., Rousselle P. (2015). How do epidermal matrix metalloproteinases support re-epithelialization during skin healing?. Artic. Eur. J. Dermatol..

[53] Pastar I., Stojadinovic O., Yin N.C., Ramirez H., Nusbaum A.G., Sawaya A., Patel S.B., Khalid L., Isseroff R.R., Tomic-Canic M. (2014). Epithelialization in Wound Healing: A Comprehensive Review. Adv. Wound Care.

[54] Krzyszczyk P., Schloss R., Palmer A., Berthiaume F. (2018). The Role of Macrophages in Acute and Chronic Wound Healing and Interventions to Promote Pro-wound Healing Phenotypes. Front. Physiol..

[55] Wynn T.A., Vannella K.M. (2016). Macrophages in Tissue Repair, Regeneration, and Fibrosis. Immunity.

[56] Martinez F.O., Gordon S. (2014). The M1 and M2 paradigm of macrophage activation: Time for reassessment. F1000Prime Rep..

[57] Schnoor M., Cullen P., Lorkowski J. (2008). Production of type VI collagen by human macrophages: A new dimension in macrophage functional heterogeneity. J. Immunol..

[58] Weitkamp B., Cullen P., Plenz G., Robenek H., Rauterberg J. (1999). Human macrophages synthesize type VIII collagen in vitro and in the atherosclerotic plaque. FASEB J..

[59] Ogle M.E., Segar C.E., Sridhar S., Botchwey E.A. (2016). Monocytes and macrophages in tissue repair: Implications for immunoregenerative biomaterial design. Exp. Biol. Med..

[60] Recalcati S., Locati M., Marini A., Santambrogio P., Zaninotto F., De Pizzol M., Zammataro L., Girelli D., Cairo G. (2010). Differential regulation of iron homeostasis during human macrophage polarized activation. Eur. J. Immunol..

[61] Mantovani A., Sica A., Sozzani S., Allavena P. (2004). The chemokine system in diverse forms of macrophage activation and polarization. Trends Immonology.

[62] Murray P.J., Wynn T.A. (2011). Protective and pathogenic functions of macrophage subsets. Nat. Rev. Immunol..

[63] Zhu Y., Li X., Chen J., Chen T., Shi Z. (2016). The pentacyclic triterpene Lupeol switches M1 macrophages to M2 and ameliorates experimental inflammatory bowel disease. Int. Immunopharmacol..

[64] Ferrante C.J., Leibovich S.J. (2012). Regulation of Macrophage Polarization and Wound Healing. Adv. Wound Care.

[65] Martinez F.O., Helming L., Gordon S. (2008). Alternative Activation of Macrophages: An Immunologic Functional Perspective. Annu. Rev. Immunol..

[66] Wang L.X., Zhang S.X., Wu H.J., Rong X.L., Guo J. (2019). M2b macrophage polarization and its roles in diseases. J. Leukoc. Biol..

[67] Das A., Sinha M., Datta S., Abas M., Chaffee S., Sen C.K., Roy S. (2015). Monocyte and Macrophage Plasticity in Tissue Repair and Regeneration. Am. J. Pathol..

[68] Larouche J., Sheoran S., Maruyama K., Martino M.M. (2018). Immune regulation of skin wound healing: Mechanisms and novel therapeutic targets. Adv. Wound Care.

[69] Zhao R., Liang H., Clarke E., Jackson C., Xue M. (2016). Inflammation in Chronic Wounds. Int. J. Mol. Sci..

[70] Dinarello C.A. (2018). Introduction to the interleukin-1 family of cytokines and receptors: Drivers of innate inflammation and acquired immunity. Immunol. Rev..

[71] Frykberg R.G., Banks J. (2015). Challenges in the Treatment of Chronic Wounds. Adv. Wound Care.

[72] Wysocki A., Staiano-Coico L. (1993). Wound fluid from chronic leg ulcers contains elevated levels of metalloproteinases MMP-2 and MMP-9. J. Investig. Dermatol..

[73] Wallace H. (1998). Levels of tumor necrosis factor-α (TNF-α) and soluble TNF receptors in chronic venous leg ulcers–correlations to healing status. J. Investig. Dermatol..

[74] Takeo M., Lee W., Ito M., Perelman R. (2015). Wound Healing and Skin Regeneration. Cold Spring Harb. Perspect Med..

[75] Jin Q., Gui L., Niu F., Yu B., Lauda N., Liu J. (2018). Macrophages in keloid are potent at promoting the differentiation and function of regulatory T cells. Exp. Cell Res..

[76] Li X., Wang Y., Yuan B. (2017). Status of M1 and M2 type macrophages in keloid. Int. J. Clin. Exp. Pathol..

[77] Ellis S., Lin E.J., Tartar D. (2018). Immunology of Wound Healing. Curr. Dermatol. Rep..

[78] Ostuni R., Kratochvill F., Murray P. (2015). Macrophages and cancer: From mechanisms to therapeutic implications. Trends Immunol..

[79] Cromack D.T., Sporn M.B., Roberts A.B., Merino M.J., Dart L.L., Norton J.A. (1987). Transforming growth factor beta levels in rat wound chambers. J. Surg. Res..

[80] Yamakawa S., Hayashida K. (2019). Advances in surgical applications of growth factors for wound healing. Burn. Trauma.

[81] Yoshida S., Yamaguchi Y., Itami S., Yoshikawa K., Tabata Y., Matsumoto K., Nakamura T. (2003). Neutralization of hepatocyte growth factor leads to retarded cutaneous wound healing associated with decreased neovascularization and granulation tissue formation. J. Investig. Dermatol..

[82] Wang Y., Weil B.R., Herrmann J.L., Abarbanell A.M., Tan J., Markel T.A., Kelly M.L., Meldrum D.R. (2009). MEK, p38, and PI-3K mediate cross talk between EGFR and TNFR in enhancing hepatocyte growth factor production from human mesenchymal stem cells. Am. J. Physiol. Cell Physiol..

[83] Joseph P., Christopher C. Skin Grafting -StatPearls -NCBI Bookshelf. https://www.ncbi.nlm.nih.gov/books/NBK532874/.

[84] Immunobiology-NCBI Bookshelf, (n.d.). https://www.ncbi.nlm.nih.gov/books/NBK10757/.

[85] Middelkoop E. (2018). Skin substitutes and “the next level”. Total Burn Care.

[86] Hardin-Young J., Teumer J., Ross R.N. (2020). Approaches to transplanting engineered cells and tissues. Princ. Tissue Eng..

[87] Buchbinder D. (2007). Wound healing: Adjuvant therapy and treatment adherence. Venous Ulcers.

[88] Carter J.E., Holmes J.H. (2016). The Surgical Management of Burn Wounds. Skin Tissue Engineering and Regenerative Medicine.

[89] Cascalho M. (2008). Challenges and potentials of xenotransplantation. Clin. Immunol..

[90] Kuo S., Kim H.M., Wang Z., Bingham E.L., Miyazawa A., Marcelo C.L., Feinberg S.E. (2018). Comparison of two decellularized dermal equivalents. J. Tissue Eng. Regen. Med..

[91] Bello Y.M., Falabella A.F., Eaglstein W.H. (2001). Tissue-engineered skin: Current status in wound healing. Am. J. Clin. Dermatol..

[92] Pourmoussa A., Gardner D.J., Johnson M.B., Wong A.K. (2016). An update and review of cell-based wound dressings and their integration into clinical practice. Ann. Transl. Med..

[93] Becker D.A.J., McCulloch E.A., Till J.E. (1963). Cytological Demonstration of the Clonal Nature of Spleen Colonies Derived from Transplanted Mouse Marrow Cells. Nature.

[94] Siminovitch L., McCulloch E.A., Till J.E. (1963). The Distribution of Colony-Forming Cells among Spleen Colonies. J. Cell. Comp. Physiol..

[95] Duscher D., Barrera J., Wong V., Maan Z. (2016). Stem cells in wound healing: The future of regenerative medicine? A mini-review. Gerontology.

[96] Gorecka J., Kostiuk V., Fereydooni A., Gonzalez L., Luo J., Dash B., Isaji T., Ono S., Liu S., Lee S.R. (2019). The potential and limitations of induced pluripotent stem cells to achieve wound healing. Stem Cell Res. Ther..

[97] Dash B., Xu Z., Lin L., Koo A., Ndon S., Berthiaume F., Dardik A., Hsia H. (2018). Stem Cells and Engineered Scaffolds for Regenerative Wound Healing. Bioengineering.

[98] Smith A.G. (2001). Embryo-derived stem cells: Of mice and men. Annu. Rev. Cell Dev. Biol..

[99] Martin G.R. (1981). Isolation of a pluripotent cell line from early mouse embryos cultured in medium conditioned by teratocarcinoma stem cells. Proc. Natl. Acad Sci. USA.

[100] Medvedev S.P., Shevchenko A.I., Zakian S.M. (2010). Induced pluripotent stem cells: Problems and advantages when applying them in regenerative medicine. Acta Nat..

[101] Aoi T., Yae K., Nakagawa M., Ichisaka T., Okita K., Takahashi K., Chibaand T., Yamanaka S. (2008). Generation of pluripotent stem cells from adult mouse liver and stomach cells. Science.

[102] Hanna J., Markoulaki S., Schorderet P. (2008). Direct reprogramming of terminally differentiated mature B lymphocytes to pluripotency. Cell.

[103] Kim J.B., Zaehres H., Wu G. (2014). Pluripotent stem cells induced from adult neural stem cells by reprogramming with two factors, nature.com. Nature.

[104] Eminli S., Utikal J., Arnold K., Jaenisch R., Hochedlinger K. (2008). Reprogramming of Neural Progenitor Cells into Induced Pluripotent Stem Cells in the Absence of Exogenous Sox2 Expression. Stem Cells.

[105] Takahashi K., Tanabe K., Ohnuki M., Narita M. (2007). Induction of pluripotent stem cells from adult human fibroblasts by defined factors. Cell.

[106] Yu J., Vodyanik M.A., Smuga-Otto K., Antosiewicz-Bourget J., Frane J.L., Tian S., Nie J., Jonsdottir G.A., Ruotti V., Stewart R. (2007). Induced Pluripotent Stem Cell Lines Derived from Human Somatic Cells. Science.

[107] Lian Q., Liang X., Ding Y., Zhang Y., Tse H.-F. (2014). Paracrine Mechanisms of Mesenchymal Stem Cell-Based Therapy: Current Status and Perspectives. Cell Transplant..

[108] Baraniak P.R., McDevitt T.C. (2010). Stem cell paracrine actions and tissue regeneration. Regen. Med..

[109] Açikgoz G., Devrim İ., Özdamar Ş. (2004). Comparison of Keratinocyte Proliferation in Diabetic and Non-Diabetic Inflamed Gingiva. J. Periodontol..

[110] Kim K.L., Song S.H., Choi K.S., Suh W. (2013). Cooperation of endothelial and smooth muscle cells derived from human induced pluripotent stem cells enhances neovascularization in dermal wounds. Proceedings of the Tissue Engineering—Part A.

[111] Clayton Z., Tan R. (2018). Induced pluripotent stem cell-derived endothelial cells promote angiogenesis and accelerate wound closure in a murine excisional wound healing model. Biosci. Rep..

[112] Casqueiro J., Casqueiro J. (2012). Infections in patients with diabetes mellitus: A review of pathogenesis. Indian J. Endocrinol. Metab..

[113] Zhang J., Guan J., Niu X., Hu G., Guo S., Li Q., Xie Z., Zhang C., Wang Y. (2015). Exosomes released from human induced pluripotent stem cells-derived MSCs facilitate cutaneous wound healing by promoting collagen synthesis and angiogenesis. J. Transl. Med..

[114] Itoh M., Umegaki-Arao N., Guo Z., Liu L., Higgins C.A., Christiano A.M. (2013). Generation of 3D Skin Equivalents Fully Reconstituted from Human Induced Pluripotent Stem Cells (iPSCs). PLoS ONE.

[115] Kuzuya M., Satake S., Esaki T., Yamada K., Hayashi T., Naito M., Asai K., Iguchi A. (1995). Induction of angiogenesis by smooth muscle cell-derived factor: Possible role in neovascularization in atherosclerotic plaque. J. Cell. Physiol..

[116] Shen Y.I., Cho H., Papa A.E., Burke J.A., Chan X.Y., Duh E.J., Gerecht S. (2016). Engineered human vascularized constructs accelerate diabetic wound healing. Biomaterials.

[117] Tan R.P., Chan A.H.P., Lennartsson K., Miravet M.M., Lee B.S.L., Rnjak-Kovacina J., Clayton Z.E., Cooke J.P., Ng M.K.C., Patel S. (2018). Integration of induced pluripotent stem cell-derived endothelial cells with polycaprolactone/gelatin-based electrospun scaffolds for enhanced therapeutic angiogenesis. Stem Cell Res. Ther..

[118] Kashpur O., Smith A., Gerami-Naini B., Maione A.G., Calabrese R., Tellechea A., Theocharidis G., Liang L., Pastar I., Tomic-Canic M. (2019). Differentiation of diabetic foot ulcer-derived induced pluripotent stem cells reveals distinct cellular and tissue phenotypes. FASEB J..

[119] Nakayama C., Fujita Y., Matsumura W., Ujiie I., Takashima S., Shinkumaa S., Nomura T., Abe R., Shimizu H. (2018). The development of induced pluripotent stem cell-derived mesenchymal stem/stromal cells from normal human and RDEB epidermal keratinocytes. J. Dermatol. Sci..

[120] Kobayashi H. (2018). Effects of Exosomes Derived from the Induced Pluripotent Stem Cells on Skin Wound Healing.

[121] Liubaviciute A., Ivaskiene T., Biziuleviciene G. (2020). Modulated mesenchymal stromal cells improve skin wound healing. Biologicals.

[122] Tencerova M., Kassem M. (2016). The bone marrow-derived stromal cells: Commitment and regulation of adipogenesis. Front. Endocrinol..

[123] Lecka-Czernik B., Rosen C.J. (2015). Energy Excess, Glucose Utilization, and Skeletal Remodeling: New Insights. J. Bone Miner. Res..

[124] Lindner U., Kramer J., Rohwedel J., Schlenke P. (2010). Mesenchymal Stem or Stromal Cells: Toward a Better Understanding of Their Biology?. Transfus Med. Hemother..

[125] Abboud S. (1993). A bone marrow stromal cell line is a source and target for platelet-derived growth factor. Blood.

[126] Rocha B., Calamia V., Mateos J., Fernández-Puente P., Blanco F.J., Ruiz-Romero C. (2012). Metabolic labeling of human bone marrow mesenchymal stem cells for the quantitative analysis of their chondrogenic differentiation. J. Proteome Res..

[127] Wu Y., Wang J.F., Scott P.G., Tredget E.E. (2007). Bone marrow-derived stem cells in wound healing: A review. Wound Repair Regen..

[128] Hao L., Wang J., Zou Z., Yan G., Dong S., Deng J. (2009). Transplantation of BMSCs expressing hPDGF-A/hBD2 promotes wound healing in rats with combined radiation-wound injury. Gene Ther..

[129] Basiouny H., Salama N. (2013). Effect of bone marrow derived mesenchymal stem cells on healing of induced full-thickness skin wounds in albino rat. Int. J. Stem Cells.

[130] Wu Y., Chen L., Scott P.G., Tredget E.E. (2007). Mesenchymal Stem Cells Enhance Wound Healing Through Differentiation and Angiogenesis. Stem Cells.

[131] Dash N.R., Dash S.N., Routray P., Mohapatra S., Mohapatra P.C. (2009). Targeting nonhealing ulcers of lower extremity in human through autologous bone marrow-derived mesenchymal stem cells. Rejuvenation Res..

[132] Sorrell J.M., Caplan A.I. (2010). Topical delivery of mesenchymal stem cells and their function in wounds. Stem Cell Res. Ther..

[133] Orlic D., Kajstura J., Chimenti S. (2001). Bone marrow cells regenerate infarcted myocardium. Nature.

[134] Horwitz E.M., Prockop D.J., Fitzpatrick L.A., Koo W.W.K., Gordon P.L., Neel M., Sussman M., Orchard P., Marx J.C., Pyeritz R.E. (1999). Transplantability and therapeutic effects of bone marrow-derived mesenchymal cells in children with osteogenesis imperfecta. Nat. Med..

[135] Salinas C.N., Anseth K.S. (2009). Mesenchymal stem cells for craniofacial tissue regeneration: Designing hydrogel delivery vehicles. J. Dent. Res..

[136] Lei Z., Singh G., Min Z., Shixuan C. (2018). Bone marrow-derived mesenchymal stem cells laden novel thermo-sensitive hydrogel for the management of severe skin wound healing. Mater. Sci. Eng. C Mater. Biol. Appl..

[137] Viezzer C., Mazzuca R., Machado D.C. (2020). A new waterborne chitosan-based polyurethane hydrogel as a vehicle to transplant bone marrow mesenchymal cells improved wound healing of ulcers in a diabetic. Carbohydr. P.

[138] Bharti M., Bhat I., Pandey S., Shabir U., Peer B.A., Indu B., Abas Rashid Bhat G.S.K., Sharma G.T. (2020). Effect of cryopreservation on therapeutic potential of canine bone marrow derived mesenchymal stem cells augmented mesh scaffold for wound healing in guinea pig. Biomed. Pharmacother..

[139] Erben R., Scutt A., Miao D. (1997). Short-Term Treatment of Rats with High Dose 1,25-Dihydroxyvitamin D3 Stimulates Bone Formation and Increases the Number of Osteoblast Precursor Cells in Bone. Endocrinology.

[140] Yoshimura K., Shigeura T., Matsumoto D., Sato T., Takaki Y., Aiba-Kojima E., Sato K., Inoue K., Nagase T., Koshima I. (2006). Characterization of freshly isolated and cultured cells derived from the fatty and fluid portions of liposuction aspirates. J. Cell. Physiol..

[141] Conese M., Annacontini L., Carbone A., Beccia E., Cecchino L.R., Parisi D., Gioia S., Di Lembo F., Angiolillo A., Mastrangelo F. (2020). The Role of Adipose-Derived Stem Cells, Dermal Regenerative Templates, and Platelet-Rich Plasma in Tissue Engineering-Based Treatments of Chronic Skin Wounds. Stem Cells Int..

[142] Patrikoski M., Mannerström B. (2019). Perspectives for clinical translation of adipose stromal/stem cells. Stem Cells Int..

[143] Ntege E., Sunami H. (2020). Advances in regenerative therapy: A review of the literature and future directions. Regen. Ther..

[144] Peng Q., Alipour H., Porsborg S., Fink T., Zachar V. (2020). Evolution of ASC Immunophenotypical Subsets During Expansion In Vitro. Int. J. Mol. Sci. Artic..

[145] Mohamed-Ahmed S., Fristad I., Lie S.A., Suliman S., Mustafa K., Vindenes H., Idris S.B. (2018). Adipose-derived and bone marrow mesenchymal stem cells: A donor-matched comparison. Stem Cell Res. Ther..

[146] Yu G., Wu X., Dietrich M.A., Polk P., Scott L.K., Ptitsyn A.A., Gimble J.M. (2010). Yield and characterization of subcutaneous human adipose-derived stem cells by flow cytometric and adipogenic mRNA analyzes. Cytotherapy.

[147] Gimble J.M., Katz A.J., Bunnell B.A. (2007). Adipose-derived stem cells for regenerative medicine. Circ. Res..

[148] Gimble J., Design M.N. (2011). Adipose-derived stromal/stem cells (ASC) in regenerative medicine: Pharmaceutical applications. Curr. Pharm. Des..

[149] Rodriguez J., Pratta A., Abbassi N., Fabre H. (2017). Evaluation of three devices for the isolation of the stromal vascular fraction from adipose tissue and for ASC culture: A comparative study. Stem Cells Int..

[150] Hur W., Lee H.Y., Min H.S., Wufuer M., Lee C.W., Hur J.A., Kim S.H., Kim B.K., Choi T.H. (2017). Regeneration of full-thickness skin defects by differentiated adipose-derived stem cells into fibroblast-like cells by fibroblast-conditioned medium. Stem Cell Res. Ther..

[151] Kim W.S., Park B.S., Sung J.H., Yang J.M., Park S.B., Kwak S.J., Park J.S. (2007). Wound healing effect of adipose-derived stem cells: A critical role of secretory factors on human dermal fibroblasts. J. Dermatol. Sci..

[152] Lee H.C., An S.G., Lee H.W., Park J.-S., Cha K.S., Hong T.J., Park J.H., Lee S.Y., Kim S.-P., Kim Y.D. (2012). Safety and Effect of Adipose Tissue-Derived Stem Cell Implantation in Patients With Critical Limb Ischemia. Circ. J..

[153] Bura A., Planat-Benard V., Bourin P. (2014). Phase I trial: The use of autologous cultured adipose-derived stroma/stem cells to treat patients with non-revascularizable critical limb ischemia. Cytotherapy.

[154] Parvizi M., Harmsen M.C. (2015). Therapeutic Prospect of Adipose-Derived Stromal Cells for the Treatment of Abdominal Aortic Aneurysm. Stem Cells Dev..

[155] Rennert R.C., Sorkin M., Januszyk M., Duscher D., Kosaraju R., Chung M.T., Lennon J., Radiya-Dixit A., Raghvendra S., Maan Z.N. (2014). Diabetes impairs the angiogenic potential of adipose-derived stem cells by selectively depleting cellular subpopulations. Stem Cell Res. Ther..

[156] Siennicka K., Zolocinska A., Stepien K., Lubina-Dabrowska N., Maciagowska M., Zolocinska E., Slysz A., Piusinska-Macoch R., Mazur S., Zdanowicz U. (2016). Adipose-Derived Cells (Stromal Vascular Fraction) Transplanted for Orthopedical or Neurological Purposes: Are They Safe Enough?. Stem Cells Int..

[157] Kim S.W., Choi J.W., Lee C.Y., Lee J., Shin S., Lim S., Lee S., Kim I.K., Lee H.B., Hwang K.C. (2018). Effects of donor age on human adipose-derived adherent stromal cells under oxidative stress conditions. J. Int. Med. Res..

[158] Klar A.S., Michalak-Mićka K., Biedermann T., Simmen-Meuli C., Reichmann E., Meuli M. (2018). Characterization of M1 and M2 polarization of macrophages in vascularized human dermo-epidermal skin substitutes in vivo. Pediatr. Surg. Int..

[159] Barsotti M.C., Magera A., Armani C., Chiellini F., Felice F., Dinucci D., Piras A.M., Minnocci A., Solaro R., Soldani G. (2011). Fibrin acts as biomimetic niche inducing both differentiation and stem cell marker expression of early human endothelial progenitor cells. Cell Prolif..

[160] Davis H., Miller S., Case E.M., Leach J.K. (2011). Supplementation of fibrin gels with sodium chloride enhances physical properties and ensuing osteogenic response. Acta Biomater..

[161] Murphy K., Whitehead J., Zhou D., Ho S.S., Leach J.K. (2017). Engineering fibrin hydrogels to promote the wound healing potential of mesenchymal stem cell spheroids. Acta Biomater..

[162] Chae D., Han S., Son M. (2017). Stromal vascular fraction shows robust wound healing through high chemotactic and epithelialization property. Cytotherapy.

[163] Gobin A.S., West J.L. (2003). Effects of Epidermal Growth Factor on Fibroblast Migration through Biomimetic Hydrogels. Biotechnol. Prog..

[164] Blay J., Brown K.D. (1985). Epidermal growth factor promotes the chemotactic migration of cultured rat intestinal epithelial cells. J. Cell. Physiol..

[165] Matthay M., Thiery J., Lafont F. (1993). Transient effect of epidermal growth factor on the motility of an immortalized mammary epithelial cell line. J. Cell. Sci..

[166] Nilforoushzadeh M.A., Sisakht M.M., Amirkhani M.A., Seifalian A.M., Banafshe H.R., Verdi J., Nouradini M. (2020). Engineered skin graft with stromal vascular fraction cells encapsulated in fibrin–collagen hydrogel: A clinical study for diabetic wound healing. J. Tissue Eng. Regen. Med..

[167] Guo J., Hu H., Gorecka J., Bai H., He H., Assi R., Isaji T., Wang T., Setia O., Lopes L. (2018). Adipose-derived mesenchymal stem cells accelerate diabetic wound healing in a similar fashion as bone marrow-derived cells. Am. J. Physiol.Cell Physiol..

[168] De Ugarte D., Morizono K., Elbarbary A. (2003). Comparison of multi-lineage cells from human adipose tissue and bone marrow. Cells Tissues Organs.

[169] Fromm-Dornieden C., Koenen P. (2013). Adipose-Derived Stem Cells in Wound Healing: Recent Results In Vitro and In Vivo. OA Mol. Cell Biol..

[170] Aggarwal S., Pittenger M.F. (2004). Human mesenchymal stem cells modulate allogeneic immune cell responses. Blood.

[171] di Nicola M., Carlo-Stella C., Magni M., Milanesi M., Longoni P.D., Matteucci P., Grisanti S., Gianni A.M. (2002). Human bone marrow stromal cells suppress T-lymphocyte proliferation induced by cellular or nonspecific mitogenic stimuli. Blood.

[172] Castro-Manrreza M., Montesinos J. (2015). Immunoregulation by Mesenchymal Stem Cells: Biological Aspects and Clinical Applications. J. Immunol. Res..

[173] Krampera M., Glennie S., Dyson J., Scott D., Laylor R., Simpson E., Dazzi F. (2003). Bone marrow mesenchymal stem cells inhibit the response of naive and memory antigen-specific T cells to their cognate peptide. Blood.

[174] Meisel R., Zibert A., Laryea M., Göbel U., Däubener W., Dilloo D., Gö U., Dä W. (2004). Human bone marrow stromal cells inhibit allogeneic T-cell responses by indoleamine 2,3-dioxygenase-mediated tryptophan degradation. Blood.

[175] Rhijn M.R., Reinders M. (2012). Mesenchymal stem cells derived from adipose tissue are not affected by renal disease. Kidney Int..

[176] Tögel F. (2010). Mesenchymal stem cells: A new therapeutic tool for AKI. Nat. Rev. Nephrol..

[177] Rahimnejad M., Derakhshanfar S., Zhong W. (2017). Biomaterials and tissue engineering for scar management in wound care. Burn. Trauma.

[178] Li Z., Wang H., Yang B., Sun Y. (2015). Three-dimensional graphene foams loaded with bone marrow derived mesenchymal stem cells promote skin wound healing with reduced scarring. Mater. Sci. Eng. C Mater. Biol. Appl..

[179] Maggini J., Mirkin G., Bognanni I., Holmberg J., Piazzón I.M., Nepomnaschy I., Costa H., Cañones C., Raiden S., Vermeulen M. (2010). Mouse bone marrow-derived mesenchymal stromal cells turn activated macrophages into a regulatory-like profile. PLoS ONE.

[180] Jackson W.M., Nesti L.J., Tuan R.S. (2012). Mesenchymal stem cell therapy for attenuation of scar formation during wound healing. Stem Cell Res. Ther..

[181] Moore K.W., De Waal Malefyt R., Coffman R.L., O’Garra A. (2001). Interleukin-10 and the interleukin-10 receptor. Annu. Rev. Immunol..

[182] Liechty K., Kim H., Adzick N.S. (2000). Fetal wound repair results in scar formation in interleukin-10–deficient mice in a syngeneic murine model of scarless fetal wound repair. J. Pediatr. Surg..

[183] Peranteau W., Zhang L., Muvarak N., Badillo A.T. (2008). IL-10 overexpression decreases inflammatory mediators and promotes regenerative healing in an adult model of scar formation. J. Investig. Dermatol.

[184] Gordon A., Kozin E.D., Keswani S.G., Vaikunth S.S., Katz A.B., Zoltick P.W., Favata M., Radu A.P., Soslowsky L.J., Herlyn M. (2008). Permissive environment in postnatal wounds induced by adenoviral-mediated overexpression of the anti-inflammatory cytokine interleukin-10 prevents scar formation. Wound Repair Regen..

[185] Mattar P., Bieback K. (2015). Comparing the immunomodulatory properties of bone marrow, adipose tissue, and birth-associated tissue mesenchymal stromal cells. Front. Immunol..

[186] Ceccarelli S., Pontecorvi P. (2020). Immunomodulatory Effect of Adipose-Derived Stem Cells: The Cutting Edge of Clinical Application. Front. Cell Dev. Biol..

[187] Cao C., Tarlé S., Kaigler D. (2020). Characterization of the immunomodulatory properties of alveolar bone-derived mesenchymal stem cells. Stem Cell Res. Ther..

[188] Rostami Z., Khorashadizadeh M., Letters M.N. (2020). Immunoregulatory properties of mesenchymal stem cells: Micro-RNAs. Immunol. Lett..

[189] Wang M., Xie J., Wang C., Zhong D. (2020). Immunomodulatory Properties of Stem Cells in Periodontitis: Current Status and Future Prospective. Stem Cell Int..

[190] Gonzalez-Rey E., Anderson P., González M. (2009). Human adult stem cells derived from adipose tissue protect against experimental colitis and sepsis. Gut.

[191] Hong S., Traktuev D., Organ K.M. (2010). Therapeutic potential of adipose-derived stem cells in vascular growth and tissue repair. Curr. Opin. Organ. Transplant..

[192] Mitchell J.B., McIntosh K., Zvonic S., Garrett S., Floyd Z.E., Kloster A., Di Halvorsen Y., Storms R.W., Goh B., Kilroy G. (2006). Immunophenotype of Human Adipose-Derived Cells: Temporal Changes in Stromal-Associated and Stem Cell-Associated Markers. Stem Cells.

[193] Puissant B., Barreau C., Bourin P., Clavel C., Corre J., Bousquet C., Taureau C., Cousin B., Abbal M., Laharrague P. (2005). Immunomodulatory effect of human adipose tissue-derived adult stem cells: Comparison with bone marrow mesenchymal stem cells. Br. J. Haematol..

[194] Wolbank S., Peterbauer A., Fahrner M., Hennerbichler S., Van Griensven M., Stadler G., Redl H., Gabriel C. (2007). Dose-dependent immunomodulatory effect of human stem cells from amniotic membrane: A comparison with human mesenchymal stem cells from adipose tissue. Tissue Eng..

[195] Yoo K., Jang I., Lee M., Kim H., Yang M. (2009). Comparison of immunomodulatory properties of mesenchymal stem cells derived from adult human tissues. Cell Immunol..

[196] Lindroos B., Suuronen R., Miettinen S. (2011). The Potential of Adipose Stem Cells in Regenerative Medicine. Stem Cell Rev. Reports.

[197] Kucerova L., Altanerova V., Matuskova M., Tyciakova S., Altaner C. (2007). Adipose Tissue-Derived Human Mesenchymal Stem Cells Mediated Prodrug Cancer Gene Therapy. Cancer Res..

[198] Yu J.M., Jun E.S., Bae Y.C., Jung J.S. (2008). Mesenchymal stem cells derived from human adipose tissues favor tumor cell growth in vivo. Stem Cells Dev..

[199] Barone A.L., Khalifian S., Lee W.P. (2013). Immunomodulatory effects of adipose-derived stem cells: Fact or fiction?. Biomed. Res. Int..

[200] Cui L., Shuo Y., Liu W., Li N., Zhang W., Cao Y. (2007). Expanded adipose-derived stem cells suppress mixed lymphocyte reaction by secretion of prostaglandin E2. Tissue Eng..

[201] Lin C.S., Lin G., Lue T.F. (2012). Allogeneic and xenogeneic transplantation of adipose-derived stem cells in immunocompetent recipients without immunosuppressants. Stem Cells Dev..

[202] Gonzalez-Rey E., Gonzalez M.A., Varela N., O’Valle F., Hernandez-Cortes P., Rico L., Büscher D., Delgado M. (2010). Human adipose-derived mesenchymal stem cells reduce inflammatory and T cell responses and induce regulatory T cells in vitro in rheumatoid arthritis. Ann. Rheum. Dis..

[203] Fang B., Song Y., Liao L., Zhang Y. (2007). Favorable response to human adipose tissue-derived mesenchymal stem cells in steroid-refractory acute graft-versus-host disease. Transpl. Proc..

[204] Fang B., Song Y., Lin Q., Zhang Y., Cao Y., Zhao R.C., Ma Y. (2007). Human adipose tissue-derived mesenchymal stromal cells as salvage therapy for treatment of severe refractory acute graft-vs.-host disease in two children. Pediatr. Transplant..

[205] Fang B., Song Y., Zhao R., Han Q. (2007). Using human adipose tissue-derived mesenchymal stem cells as salvage therapy for hepatic graft-versus-host disease resembling acute hepatitis. Transpl. Proc..

[206] Park H., Cho J., Lim E., Lee C., Lee S. (2011). Cell cycle regulators are critical for maintaining the differentiation potential and immaturity in adipogenesis of adipose-derived stem cells. Differentiation.

[207] Jiang W., Xu J. (2020). Immune modulation by mesenchymal stem cells. Cell Prolif..

[208] Bartholomew A., Sturgeon C., Siatskas M., Ferrer K., McIntosh K., Patil S., Hardy W., Devine S., Ucker D., Deans R. (2002). Mesenchymal stem cells suppress lymphocyte proliferation in vitro and prolong skin graft survival in vivo. Exp. Hematol..

[209] Koppula P.R., Chelluri L.K., Polisetti N., Vemuganti G.K. (2009). Histocompatibility testing of cultivated human bone marrow stromal cells—A promising step towards pre-clinical screening for allogeneic stem cell therapy. Cell. Immunol..

[210] Mohanty A., Polisetti N., Vemuganti G.K. (2020). Immunomodulatory properties of bone marrow mesenchymal stem cells. J. Biosci..

[211] Bertozzi N., Simonacci F. (2017). The biological and clinical basis for the use of adipose-derived stem cells in the field of wound healing. Ann. Med. Surg.

[212] Franz S., Rammelt S., Scharnweber D., Simon J.C. (2011). Immune responses to implants—A review of the implications for the design of immunomodulatory biomaterials. Biomaterials.

[213] Sun G., Shen Y.-I., Harmon J.W. (2018). Engineering Pro-Regenerative Hydrogels for Scarless Wound Healing. Adv. Healthc. Mater..

[214] Vishwakarma A., Bhise N.S., Evangelista M.B., Rouwkema J., Dokmeci M.R., Ghaemmaghami A.M., Vrana N.E., Khademhosseini A. (2016). Engineering Immunomodulatory Biomaterials To Tune the Inflammatory Response. Trends Biotechnol..

[215] Mantovani A., Biswas S.K., Galdiero M.R., Sica A., Locati M. (2013). Macrophage plasticity and polarization in tissue repair and remodelling. J. Pathol..

[216] Brown B., Ratner B., Goodman S. (2012). Macrophage polarization: An opportunity for improved outcomes in biomaterials and regenerative medicine. Biomaterials.

[217] Williams D.F. (2008). On the mechanisms of biocompatibility. Biomaterials.

[218] Li J., Hastings G.W. (2016). Oxide bioceramics: Inert ceramic materials in medicine and dentistry. Handbook of Biomaterial Properties.

[219] Hayashi K., Inadome T., Tsumura H. (1993). Bone-implant interface mechanics of in vivo bio-inert ceramics. Biomaterials.

[220] Ehashi T., Takemura T., Hanagata N., Minowa T., Kobayashi H., Ishihara K., Yamaoka T. (2014). Comprehensive genetic analysis of early host body reactions to the bioactive and bio-inert porous scaffolds. PLoS ONE.

[221] Hench L.L., Wilson J. (1993). An Introduction to Bioceramics.

[222] Desai T. (2017). Advances in islet encapsulation technologies. Nat. Rev. Drug Discov..

[223] Onuki Y., Bhardwaj U., Papadimitrakopoulos F., Burgess D.J. (2008). A review of the biocompatibility of implantable devices: Current challenges to overcome foreign body response. J. Diabetes Sci. Technol..

[224] Zakeri Siavashani A., Mohammadi J., Maniura-Weber K., Senturk B., Nourmohammadi J., Sadeghi B., Huber L., Rottmar M. (2020). Silk based scaffolds with immunomodulatory capacity: Anti-inflammatory effects of nicotinic acid. Biomater. Sci..

[225] Hench L.L., Thompson I. (2010). Twenty-first century challenges for biomaterials, Royalsocietypublishing.Org. J. R. Soc. Interface.

[226] Dziki J.L., Huleihel L., Scarritt M.E., Badylak S.F. (2017). Extracellular Matrix Bioscaffolds as Immunomodulatory Biomaterials. Tissue Eng. Part. A.

[227] Anderson J., Rodriguez A. (2008). Foreign body reaction to biomaterials. Semin. Immunol..

[228] Vallés G., Bensiamar F., Crespo L., Arruebo M. (2015). Topographical cues regulate the crosstalk between MSCs and macrophages. Biomaterials.

[229] Broughton G.I., Jeffrey Janis U.E., Attinger C.E. (2006). The Basic Science of Wound Healing. Plast Reconstr. Surg..

[230] Badylak S.F., Valentin J.E., Ravindra A.K., McCabe G.P., Stewart-Akers A.M. (2008). Macrophage phenotype as a determinant of biologic scaffold remodeling. Tissue Eng. Part. A..

[231] Brown B.N., Valentin J.E., Stewart-Akers A.M., McCabe G.P., Badylak S.F. (2009). Macrophage phenotype and remodeling outcomes in response to biologic scaffolds with and without a cellular component. Biomaterials.

[232] Koh T., Medicine L.D. (2011). Inflammation and wound healing: The role of the macrophage. Expert Rev. Mol..

[233] Gordon S. (2003). Alternative activation of macrophages. Nat. Rev. Immunol..

[234] Sridharan R., Cameron A., Kelly D. (2015). Biomaterial based modulation of macrophage polarization: A review and suggested design principles. Materialstoday.

[235] Singh S., Awuah D., Rostam H.M., Emes R.D., Kandola N.K., Onion D., Htwe S.S., Rajchagool B., Cha B.H., Kim D. (2017). Unbiased Analysis of the Impact of Micropatterned Biomaterials on Macrophage Behavior Provides Insights beyond Predefined Polarization States. ACS Biomater. Sci. Eng..

[236] Corliss B.A., Azimi M.S., Munson J.M., Peirce S.M., Murfee W.L. (2016). Macrophages: An Inflammatory Link Between Angiogenesis and Lymphangiogenesis. Microcirculation.

[237] Ashouri F., Beyranvand F., Boroujeni N.B., Tavafi M., Sheikhian A., Varzi A.M., Shahrokhi S. (2019). Macrophage polarization in wound healing: Role of aloe vera/chitosan nanohydrogel. Drug Deliv. Transl. Res..

[238] Kumar M., Gupta P., Bhattacharjee S. (2018). Immunomodulatory injectable silk hydrogels maintaining functional islets and promoting anti-inflammatory M2 macrophage polarization. Biomaterials.

[239] Landén N.X., Li D., Ståhle M. (2016). Transition from inflammation to proliferation: A critical step during wound healing. Cell. Mol. Life Sci..

[240] Lucas T., Waisman A., Ranjan R. (2010). Differential roles of macrophages in diverse phases of skin repair. J. Immunol..

[241] Klar A.S.A., Biedermann T., Simmen-Meuli C., Reichmann E., Meuli M., Simmen-Meuli C. (2017). Comparison of in vivo immune responses following transplantation of vascularized and non-vascularized human dermo-epidermal skin substitutes. Pediatr. Surg..

[242] Brown B., Londono R., Tottey S., Zhang L. (2012). Macrophage phenotype as a predictor of constructive remodeling following the implantation of biologically derived surgical mesh materials. Acta Biomater..

[243] Meng F.W., Slivka P.F., Dearth C.L., Badylak S.F. (2015). Solubilized extracellular matrix from brain and urinary bladder elicits distinct functional and phenotypic responses in macrophages. Biomaterials.

[244] Chakraborty J., Roy S., Ghosh S. (2020). Regulation of decellularized matrix mediated immune response. Biomater. Sci..

[245] Kharaziha S., Baidya A., Annabi N. (2021). Rational Design of Immunomodulatory Hydrogels for Chronic Wound Healing. Adv. Mater.

[246] jan Lebbink R., Raynal N., de Ruiter T., Bihan D.G., Richard W., Farndale L.M. (2009). Identification of multiple potent binding sites for human leukocyte associated Ig-like receptor LAIR on collagens II and III. Matrix Biol..

[247] El Masry M.S., Chaffee S., Ghatak P.D., Mathew-Steiner S.S., Das A., Higuita-Castro N., Roy S., Anani R.A., Sen C.K. (2019). Stabilized collagen matrix dressing improves wound macrophage function and epithelialization. FASEB J..

[248] Amlericani Societlj C., Inv B., Pathologv E., Brown L.F., Lanir N., McDonagh J., Tognazzi K., Dvorak A.M., Dvorak H.F. (1993). Fibroblast Migration in Fibrin Gel Matrices. Am. J. Pathol..

[249] Clark R.A. (2001). Fibrin and wound healing. Ann. N. Y. Acad. Sci..

[250] Stone R., Natesan S., Kowalczewski C.J., Mangum L.H., Clay N.E., Clohessy R.M., Carlsson A.H., Tassin D.H., Chan R.K., Rizzo J.A. (2018). Advancements in regenerative strategies through the continuum of burn care. Front. Pharmacol..

[251] Hsieh J., Smith T., Meli V., Tran T. (2017). Differential regulation of macrophage inflammatory activation by fibrin and fibrinogen. Acta Biomater..

[252] Qu Z., Chaikof E.L. (2010). Interface between hemostasis and adaptive immunity. Curr. Opin. Immunol..

[253] Knopf-Marques H., Pravda M., Wolfova L., Velebny V., Schaaf P., Vrana N.E., Lavalle P. (2016). Hyaluronic Acid and Its Derivatives in Coating and Delivery Systems: Applications in Tissue Engineering, Regenerative Medicine and Immunomodulation. Adv. Healthc. Mater..

[254] Rayahin J.E., Buhrman J.S., Zhang Y., Koh T.J., Gemeinhart R.A. (2015). High and Low Molecular Weight Hyaluronic Acid Differentially Influence Macrophage Activation. ACS Biomater. Sci. Eng..

[255] Fong D., Hoemann C.D. (2018). Chitosan immunomodulatory properties: Perspectives on the impact of structural properties and dosage. Futur. Sci. OA.

[256] Takei T., Nakahara H., Ijima H. (2012). Synthesis of a chitosan derivative soluble at neutral pH and gellable by freeze–thawing, and its application in wound care. Acta Biomater..

[257] Porporatto C., Bianco I.D., Riera C.M., Correa S.G. (2003). Chitosan induces different L L-arginine metabolic pathways in resting and inflammatory macrophages. Biochem. Biophys. Res. Commun..

[258] Moura L.I.F., Dias A.M.A., Leal E.C., Carvalho L., De Sousa H.C., Carvalho E., Biomaterialia A., Moura L.I.F., Dias A.M.A., Leal E.C. (2013). Chitosan-based dressings loaded with neurotensin-an efficient strategy to im-prove early diabetic wound healing. Acta Biomater..

[259] Fitton J.H., Stringer D.N., Karpiniec S.S. (2015). Therapies from Fucoidan: An Update. Mar. Drugs.

[260] Wijesekara I., Pangestuti R., Kim S.K. (2011). Biological activities and potential health benefits of sulfated polysaccharides derived from marine algae. Carbohydr. Polym..

[261] Kalitnik A.A., Anastyuk S.D., Sokolova E.V., Kravchenko A.O., Khasina E.I., Yermak I.M. (2015). Oligosaccharides of κ/β-carrageenan from the red alga Tichocarpus crinitus and their ability to induce interleukin 10. J. Appl. Phycol..

[262] He M., Potuck A., Zhang Y., Chu C.C. (2014). Arginine-based polyester amide/polysaccharide hydrogels and their biological response. Acta Biomater..

[263] El-Sakka A.I., Salabas E., Dinçer M., Kadioglu A. (2013). The pathophysiology of Peyronie’s disease. Arab J. Urol..

[264] He M., Sun L., Fu X., McDonough S.P., Chu C.C. (2019). Biodegradable amino acid-based poly(ester amine) with tunable immunomodulating properties and their in vitro and in vivo wound healing studies in diabetic rats’ wounds. Acta Biomater..

[265] Singh A., Peppas N.A. (2014). Hydrogels and scaffolds for immunomodulation. Adv. Mater..

[266] Vaday G.G., Lider O. (2000). Extracellular matrix moieties, cytokines, and enzymes: Dynamic effects on immune cell behavior and inflammation. J. Leukoc. Biol..

[267] Rowley A.T., Nagalla R.R., Wang S.W., Liu W.F. (2019). Extracellular Matrix-Based Strategies for Immunomodulatory Biomaterials Engineering. Adv. Healthc. Mater..

[268] Badylak S. (2008). Immune response to biologic scaffold materials. Semin. Immunol..

[269] Taraballi F., Sushnitha M., Tsao C., Bauza G., Liverani C., Shi A., Tasciotti E. (2018). Biomimetic Tissue Engineering: Tuning the Immune and Inflammatory Response to Implantable Biomaterials. Adv. Healthc. Mater..

[270] Cramer M. (2019). Extracellular matrix-based biomaterials and their influence upon cell behavior. Biomater. Eng. Cell Behav..

[271] Huleihel L., Dziki J., Bartolacci J. (2017). Macrophage phenotype in response to ECM bioscaffolds. Semin. Immunol.

[272] Abraham G.A., Murray J., Billiar K., Sullivan S.J. (2000). Evaluation of the porcine intestinal collagen layer as a biomaterial. J. Biomed. Mater. Res..

[273] Parmaksiz M., Dogan A., Odabas S., Elçin A.E., Elçin Y.M. (2016). Clinical applications of decellularized extracellular matrices for tissue engineering and regenerative medicine. Biomed. Mater..

[274] Mokhtari H., Kharaziha M., Karimzadeh F., Tavakoli S. (2019). An injectable mechanically robust hydrogel of Kappa-carrageenan-dopamine functionalized graphene oxide for promoting cell growth. Carbohydr. Polym..

[275] Tavakoli S., Kharaziha M., Nemati S., Kalateh A. (2021). Nanocomposite hydrogel based on carrageenan-coated starch/cellulose nanofibers as a hemorrhage control material. Carbohydr. Polym..

[276] Tavakoli S., Kharaziha M., Kermanpur A., Mokhtari H. (2019). Sprayable and injectable visible-light Kappa-carrageenan hydrogel for in-situ soft tissue engineering. Int. J. Biol. Macromol..

[277] Tavakoli S., Mokhtari H., Kharaziha M., Kermanpur A., Talebi A., Moshtaghian J. (2020). A multifunctional nanocomposite spray dressing of Kappa-carrageenan-polydopamine modified ZnO/L-glutamic acid for diabetic wounds. Mater. Sci. Eng. C.

[278] Yu S., Wang C., Yu J., Wang J., Lu Y., Zhang Y., Zhang X., Hu Q., Sun W., He C. (2018). Injectable Bioresponsive Gel Depot for Enhanced Immune Checkpoint Blockade. Adv. Mater..

[279] Yang P., Song H., Qin Y., Huang P., Zhang C., Kong D., Wang W. (2018). Engineering Dendritic-Cell-Based Vaccines and PD-1 Blockade in Self-Assembled Peptide Nanofibrous Hydrogel to Amplify Antitumor T-Cell Immunity. ACS Publ..

[280] Sun Z., Liang J., Dong X., Wang C., Kong D., Lv F. (2018). Injectable Hydrogels Coencapsulating Granulocyte-Macrophage Colony-Stimulating Factor and Ovalbumin Nanoparticles to Enhance Antigen Uptake Efficiency. ACS Appl. Mater. Interfaces.

[281] Zaveri T., Lewis J., Dolgova N. (2014). Integrin-directed modulation of macrophage responses to biomaterials. Biomaterials.

[282] Wilgus T. (2008). Immune cells in the healing skin wound: Influential players at each stage of repair. Pharmacol. Res..

[283] Jones J.A., Chang D.T., Meyerson H., Colton E., Il K.K., Matsuda T., Anderson J.M. (2007). Proteomic analysis and quantification of cytokines and chemokines from biomaterial surface-adherent macrophages and foreign body giant cells. J. Biomed. Mater. Res. Part. A.

[284] Curtis A., Wilkinson C. (1997). Topographical control of cells. Biomaterials.

[285] Akira S. (2004). Toll-like receptor signalling. Nat. Rev. Immunol..

[286] Zakrzewski J., Brink M., van den Brink M. (2014). Overcoming immunological barriers in regenerative medicine. Nat. Biotechnol..

[287] Babensee J.E., Paranjpe A. (2005). Differential levels of dendritic cell maturation on different biomaterials used in combination products. J. Biomed. Mater. Res. Part A.

[288] Zhou L.T.F.T. (1996). CD14+ blood monocytes can differentiate into functionally mature CD83+ dendritic cells. Proc. Natl. Acad. Sci. USA.

[289] Banchereau J., Steinman R.M. (1998). Dendritic cells and the control of immunity. Nature.

[290] jan Lebbink R., de Ruiter T., Adelmeijer J., Brenkman A.B., van Helvoort J.M., Koch M., Farndale R.W., Lisman T., Sonnenberg A., Lenting P.J. (2006). Collagens are functional, high affinity ligands for the inhibitory immune receptor LAIR-1. J. Exp. Med..

[291] Chattopadhyay S., Raines R.T. (2014). Review collagen-based biomaterials for wound healing. Biopolymers.

[292] Witherel C.E., Graney P.L., Freytes D.O., Weingarten M.S., Spiller K.L. (2016). Response of human macrophages to wound matrices in vitro. Wound Repair Regen..

[293] De Angelis B., Orlandi F., Fernandes Lopes Morais D’Autilio M., Scioli M.G., Orlandi A., Cervelli V., Gentile P. (2018). Long-term follow-up comparison of two different bi-layer dermal substitutes in tissue regeneration: Clinical outcomes and histological findings. Int. Wound J..

[294] Agrawal H., Tholpady S., Capito A., Drake D., Katz A. (2012). Macrophage phenotypes correspond with remodeling outcomes of various acellular dermal matrices. Open J. Regen. Med..

[295] Podolnikova N.P., Yakubenko V.P., Volkov G.L., Plow E.F., Ugarova T.P. (2003). Identification of a Novel Binding Site for Platelet Integrins IIb 3 (GPIIbIIIa) and 5 1 in the C-domain of Fibrinogen. J. Biol. Chem..

[296] Clark R.A.F. (1988). Wound Repair. the Molecular and Cellular Biology of Wound Repair.

[297] (2017). Local induction of lymphangiogenesis with engineered fibrin-binding VEGF-C promotes wound healing by increasing immune cell trafficking and matrix. Biomaterials.

[298] Mokarram N., Bellamkonda R.V. (2014). A perspective on immunomodulation and tissue repair. Ann. Biomed. Eng..

[299] Hashimoto T., Kojima K. (2020). Gene expression advances skin reconstruction and wound repair better on silk fibroin-based materials than on collagen-based materials. Materialia.

[300] Burdick J.A., Prestwich G.D. (2011). Hyaluronic acid hydrogels for biomedical applications. Adv. Mater..

[301] Wang H., Morales R.T.T., Cui X., Huang J., Qian W., Tong J., Chen W. (2019). A Photoresponsive Hyaluronan Hydrogel Nanocomposite for Dynamic Macrophage Immunomodulation. Adv. Healthc. Mater..

[302] Zamboni F., Vieira S., Reis R.L., Oliveira J.M., Collins M.N. (2018). The potential of hyaluronic acid in immunoprotection and immunomodulation: Chemistry, processing and function. Prog. Mater..

[303] Ruppert S., Hawn T., Arrigoni A. (2014). Tissue integrity signals communicated by high-molecular weight hyaluronan and the resolution of inflammation. Immunol Res..

[304] Saini S., Dhiman A. (2020). Immunomodulatory Properties of Chitosan: Impact on Wound Healing and Tissue Repair. Endocr Metab. Immune. Disord Drug. Targets.

[305] Dai T., Tanaka M., Huang Y.Y., Hamblin M.R. (2011). Chitosan preparations for wounds and burns: Antimicrobial and wound-healing effects. Expert Rev. Anti. Infect. Ther..

